# Copper(II)-Bis-Cyclen Intercalated Graphene Oxide as an Efficient Two-Dimensional Nanocomposite Material for Copper-Catalyzed Azide–Alkyne Cycloaddition Reaction

**DOI:** 10.3389/fchem.2021.754734

**Published:** 2022-01-07

**Authors:** Angel Green Samuel, Sowmya Subramanian, Vijaikanth Vijendran, Jebasingh Bhagavathsingh

**Affiliations:** Department of Applied Chemistry, Karunya Institute of Technology and Sciences, Coimbatore, India

**Keywords:** intercalated graphene oxide, 2D nanocomposite, click chemistry, copper(II) complexes, azide–acetylene cycloaddition, CuAAC reaction, cyclen ligand

## Abstract

We report stable and heterogeneous graphene oxide (GO)–intercalated copper as an efficient catalyst for the organic transformations in green solvents. The GO-intercalated copper(II) complex of bis(1,4,7,10-tetraazacyclododecane) [Cu(II)-bis-cyclen] was prepared by a facile synthetic approach with a high dilution technique. The as-prepared GO-Cu(II)-bis-cyclen nanocomposite was used as a click catalyst for the 1,3 dipolar Huisgen cycloaddition reaction of terminal alkyne and azide substrates. On directing a great deal of attention toward the feasibility of the rapid electron transfer rate of the catalyst in proliferating the yield of 1,2,3-triazole products, the click catalyst GO-Cu(II)-bis-cyclen nanocomposite was designed and synthesized *via* non-covalent functionalization. The presence of a higher coordination site in an efficient 2D nanocomposite promotes the stabilization of Cu(I) L-acetylide intermediate during the catalytic cycle initiated by the addition of reductants. From the XRD analysis, the enhancement in the *d*-interlayer spacing of 1.04 nm was observed due to the intercalation of the Cu(II)-bis-cyclen complex in between the GO basal planes. It was also characterized by XPS, FT-IR, RAMAN, UV, SEM, AFM, and TGA techniques. The recyclability of the heterogeneous catalyst [GO-Cu(II)-cyclen] with the solvent effect has also been studied. This class of GO-Cu(II)-bis-cyclen nanocomposite paves the way for bioconjugation of macromolecules through the click chemistry approach.

## Introduction

The Cu(I)-catalyzed [3 + 2] cycloaddition reaction between the terminal alkynes and azides, popularly known as “click reaction,” was developed by Sharpless and Meldal, which have emerged in various branches of research (Kolb et al., 2001; Meldal and Schoffelen, 2016). Due to the versatile characteristics of click chemistry, it has been considered a crucial synthetic strategy in the transformations of organic reaction, mainly in assembling the diverse functional molecules, as reported by many researchers (Kolb and Sharpless, 2003; Binder and Sachsenhofer, 2007; Döhler et al., 2012). As defined by Rostovtsev et al. (2002), the click reactions are rapid, easy to perform, unaffected by air and protic/aprotic solvents, and wide in scope, that is, tolerant of broad functionalities contributing to the products of high atom economy. Despite the success of copper-catalyzed azide–alkyne cycloaddition (CuAAC), it depicts certain demerits such as i) contamination of click products are inferred due to the use of copper(II)–based catalyst, like in few cases, copper prefers to coordinate with the available heteroatoms in the substrates such as macromolecules or macrocycles, ii) *in situ* formation of unstable Cu(I) species by the addition of the reducing agent which become the salient prerequisite in the click reaction (Meldal and Tornøe, 2008), and iii) the copper directly coordinates with the available heteroatoms in the case of macromolecules or macrocycles and thereby the efficiency is reduced. Therefore, the demand and research on recoverable variants of heterogeneous/homogenous click catalyst, rather than the reported catalyst (CuSO_4_.5H_2_O proposed by Kolb et al. (2001)) were particularly emphasized in the number of reports over few decades (Jumde et al., 2015). In order to enhance the catalytic activity of CuAAC by the stabilization of Cu(I) species, click reaction requires certain auxiliary reagents such as ligands, bases, and oxidizing/reducing agents depending on the copper source material used (Wang et al., 2016). In several CuAAC reactions, the urgency of removing the copper catalyst and other additives used during the course of the reaction is considered an ultimate goal (Pickens et al., 2017). In order to achieve the prior shortcomings, the stable, recyclable, and heterogeneous Cu catalyst devoid of additional agents is highly desirable. Herein, we report the highly stable, dispersible, recyclable, and heterogeneous copper-based GO nanocomposites [GO-Cu(II)-bis-cyclen nanocomposite] as an efficient click catalyst for the 1,3-dipolar cycloaddition of the terminal alkynes and azides without the need of base or excess of co-catalysts. Accordingly, graphene-based materials such as graphene oxide (GO) and reduced graphene oxide (*r*GO) are used as a catalyst support or catalyst in many research studies (Julkapli and Bagheri, 2015). GO is a two-dimensional sp-hybridized (Meldal and Schoffelen, 2016) carbon containing various functionalities mimicking the graphene material surface having a single layer sheet with some defects (Saxena et al., 2011). Graphene oxide consists of different oxygen-containing functional groups such as -OH, -COOH, and -C-O-C (epoxy) possessing exceptional properties on intercalation proven to enhance the electron mobility of graphene, thereby facilitating the fast electron transfer during the catalytic reactions improving its catalytic activity in CuAAC (Dreyer et al., 2010). Besides this, GO has a high specific surface area, which in turn increases the dispersion ability of the metal particles onto the surface acting as an active site of the catalyst (Smith et al., 2019). GO exhibits high chemical, thermal, optical, and electrochemical stabilities, which may boost the life span of the catalyst (Zhao et al., 2017). In addition, on intercalation, the resultant property of GO can be enhanced because the negatively charged surface of GO can be easily exploited to intercalate the catalytic material like metal/metal oxides and metal complexes (Chakravarty et al., 2015; Amirov et al., 2017). As a result of the significant properties of GO, different types of organic reactions such as coupling reactions were performed using the metal/metal oxide–intercalated graphene oxide nanomaterials as a catalyst or catalyst support (Shaabani and Afshari, 2018; Sachdeva, 2020). Among the metal-based graphene oxide nanocomposites, recently, graphene–copper or copper oxide immobilized on graphene oxide nanocomposites was used in the catalytic application of click reactions (Mandoli, 2016). Nia et al. have reported copper nanoparticles immobilized onto the graphene nanosheets used as a heterogeneous, recyclable, and reusable catalyst for Cu(I)-catalyzed [3 + 21] cycloaddition reaction (Shaygan Nia et al., 2014). Reddy et al. (2016) have reported a click synthesis of 1,2,3-triazole derivatives under green reaction conditions using graphene oxide–supported copper oxide (CuO-GO) nanocatalyst affording an excellent selectivity and yield of the desired products (Reddy et al., 2016). A copper nanoparticle–decorated three-dimensional graphene nanocomposite exhibited excellent catalytic activity in the synthesis of 1,2,3-triazoles, as reported by Dabiri et al. (2016). There are only a limited number of reports that dealt with the copper complexes supported on the graphene oxide materials. The graphene oxide/poly(vinyl imidazole) nanocomposite as heterogeneous polymeric catalyst was reported for the click synthesis of 1,2,3-triazole derivatives in excellent yields *via* one-pot three-component cycloaddition of halides, terminal alkynes, and sodium azide (Pourjavadi et al., 2015). Yek et al. reported heterogenized Cu(II) complex of 5-amino tetrazole immobilized on graphene oxide nanosheets as an efficient catalyst for the reduction of 4-nitrophenol (4-NP), rhodamine B (RhB), methylene blue (MB), nigrosin (NS), and Congo red (CR) with NaBH_4_ (Yek et al., 2020). Novel graphene oxide (GO)–tethered Cu(II) and Co(II) salen complexes were utilized as efficient catalysts in the epoxidation of styrene as reported by Li et al. (2013). Zarnegaryan et al. reported the novel graphene oxide–immobilized Cu (II) complex of 2-bis(4-aminophenylthio) ethane as an efficient catalyst for the epoxidation of olefins with *t*-butyl hydroperoxide (Zarnegaryan et al., 2016). The Cu(II) Schiff base complex immobilized on graphene oxide has been synthesized and has demonstrated its catalytic application in the green synthesis of propargylamines from aldehydes, alkynes, and amines without using any base or co-catalyst in an aqueous medium, as reported by Kumari et al. (2016). Hamed et al. have Cu(II)–metformin immobilized on graphene oxide as an efficient and recyclable catalyst for the Beckmann rearrangement (Solaiman Hamed and Mohammad Ali, 2020). In our earlier work, we have reported the highly stable copper(II) complex–intercalated GO nanocomposite as the heterogeneous copper catalyst for the CuAAC reaction and the desired 1,4-disubstituted 1,2,3-triazoles were obtained in excellent yield (Samuel et al., 2020). The aim of the present work is to develop a heterogeneous, efficient, nanomaterial catalyst, caging the copper complex over the GO surface for the usage of click reaction in the biological substrates in aqueous medium, upon the reduction of Cu(II) complex–GO composites. It is noteworthy to report that the graphene-based material has been identified to be an excellent platform for accommodating metal and metal oxides. The catalytic properties of metals and metal nanoparticles were improved when the graphene oxide (GO) was used as a catalytic support due to their increased surface area and stability (Shabestari et al., 2020).

Herein, we report the efficiency of the GO-Cu(II)-bis-cyclen nanocomposite in the CuAAC catalytic application as a click catalyst. We have successfully intercalated the Cu(II)-bis-cyclen complex onto the graphene oxide surface *via* the facile non-covalent intercalation approach. The fabricated GO-Cu(II)-bis-cyclen nanocomposite was employed as an effective heterogeneous nanocatalyst for isolating 1,4-disubstituted-1,2,3 triazole in high yield. The stability of the GO-Cu(II)-bis-cyclen nanocomposite was substantiated by its recyclability and reusability. The recyclability study of the GO-Cu(II)-bis-cyclen nanocomposite was demonstrated by performing the click reaction of phenyl acetylene and benzyl azide under optimized conditions. It is inferred that the GO-Cu(II)-bis-cyclen nanocomposite was stable, and it was recovered and remained the same for the four reaction cycles.

## Materials and Methods

### Synthesis of Cu(II)-Bis-Cyclen Complex

The Cu(II)-bis-cyclen complex was prepared by the modified method (Hirohama et al., 2005). To the solution of cyclen ligand (1 equiv.) in 20 ml of methanol, copper(II) perchlorate hexahydrate (0.5 equiv.) in 10 ml of methanol was added dropwise over the period of 1 h under argon atmosphere. The resulting solution was refluxed overnight. Then the reaction mixture was cooled, and the solvent was removed by a rotary evaporator. The purple-colored solid was washed with diethyl ether to isolate Cu(II)-bis-cyclen complex and stored in vacuum desiccators (yield 92%). The as-prepared complex was confirmed by ESI-MS and FT-IR spectroscopy.

### Intercalation of Cu^II^-Bis-Cyclen Onto the GO Planes

Graphene oxide was synthesized by the reported method (Ramesh and Bhagavathsingh, 2017). The intercalation of the Cu(II)-bis-cyclen complex into the GO nanosheets was performed by a high dilution technique. In the typical procedure, graphene oxide (1 g) was dispersed in 200 ml of water and sonicated for an hour. To the well-dispersed GO solution, the Cu(II)-bis-cyclen complex (1 g) dissolved in 50 ml of ethanol was added dropwise under vigorous stirring over a period of 12 h. Upon the intercalation, the brown dispersed solution was changed to black color. After overnight stirring, the black solid was centrifuged and washed with water followed by ethanol to remove the excess Cu(II)-bis-cyclen complex. Diethyl ether was added to remove all the residual solvents and dried at RT to isolate the free-flow black solid. The dried GO-Cu(II)-bis-cyclen nanocomposite was stored in a desiccator and used as a click catalyst (yield: 97%).

### General Procedure for Cu^II^AAC Reaction

In a round bottom flask fitted with a stopper, phenyl acetylene (1.0 mmol), organic azide (1.0 mmol), and GO-Cu(II)-bis-cyclen nanocomposite (30 mg) were added in 2 ml of *t*-butanol followed by the addition of sodium ascorbate (10 mg) dissolved in 4 ml of water. The reaction mixture was stirred at room temperature for about 60 min. The GO-based catalyst was filtered, and the resulting reaction mixture was diluted with ethyl acetate (50 ml) and water (10 fold volume) to get a biphasic layer. The organic layer was separated and dried over anhydrous sodium sulfate. The resulting solution was concentrated under reduced pressure. The crude product was purified using silica gel column chromatography (eluents: hexane:ethyl acetate, 6:4 *v/v*) to isolate the corresponding 1,4-disubstituted-1,2,3-triazole (1c-7c) in good yields (64–89%).

### General Procedure for Cyclic Voltammetry Studies of GO-Cu(II)-Bis-Cyclen Catalyst

Electrochemical measurements were performed using a CH-660C electrochemical workstation instrument. Experiments were carried out in a three-electrode cell with glassy carbon as a working electrode, a platinum wire as the counter electrode, and Ag/AgCl (NaCl saturated) as reference electrodes; 50 mM sodium chloride and 5 mM HEPES were used as a supporting electrolyte and buffer, respectively. The surface of the working electrode was prepared by polishing with 0.3 µm alumina slurry followed by brief sonication in deionized water. For the CV study, the GO-Cu(II)-bis-cyclen nanocomposite (10 mg) was grinded well with polyvinylidene fluoride (2 mg) and made into a black paste using *N*-methyl pyrrolidone (0.5 ml). The black paste of GO-Cu(II)-bis-cyclen nanocomposite was coated on a glassy carbon electrode and left to dry for 12 h. The working chamber was degassed with argon gas and the electrochemical measurements were performed at the scan rate of 50 mV (Wang et al., 2001).

### Characterization

The absorbance was recorded at 200–800 nm using a Perkin Elmer Lambda 35 UV-Vis spectrophotometer. FTIR analysis was carried out with a spectral range of 400–4,000 cm^−1^ using the IR-Prestise-21 Shimadzu instrument. The crystalline structure of the GO and GO-Cu(II)-bis-cyclen nanocomposite was studied using the X-ray diffraction technique. The XRD patterns were recorded on a Shimadzu XRD-6,000 Powder X-Ray diffractometer at 40 kV voltage and 30 mA current. All the spectra were acquired at an atmospheric pressure using an ultra-high vacuum with Al Kα excitation at 250 W. A scanning electron microscope (SEM) images were measured by the Hitachi S4800 field emission SEM system. X-ray photoelectron spectroscopic analysis was carried out in Axis Ultra multi-technique X-ray photoelectron spectroscopy. All the spectra were acquired at an atmospheric pressure using an ultra-high vacuum with Al Kα excitation at 250 W. Raman spectra were recorded on a Horiba-Jobin Raman spectrometer with a 514-nm laser power. TGA measurements were carried out under a N_2_ atmosphere using NETZSCH STA 449 F3 Jupiter. ^1^H and ^13^C-NMR spectra of click products were recorded with a Bruker Avance III HD Nanobay 400 MHz FT-NMR Spectrometer. The mass spectrum of the Cu(II)-bis-cyclen-complex and triazole compounds were recorded in Agilent mass spectrometer LCMS-1260 INFINITY II under a nitrogen atmosphere. An Agilent 1260 mass spectrometer was employed for ESI-MS analysis at 17 eV scan mode. Electrochemical measurements were performed using a CH-660C electrochemical workstation with a three-electrode system. The surface morphology of the samples was studied using AFM (M/S NT-MDT, along with NOVA-PX software) in semi-contact mode. All chemicals and solvents were purchased from commercial suppliers such as Aldrich, TCI, and Himedia. The azides were prepared from the corresponding halides treated with sodium azide in dry acetone (Patai and Rappoport, 1995).

## Result and Discussion

### Intercalated Material Characterization

**FIGURE 1:**
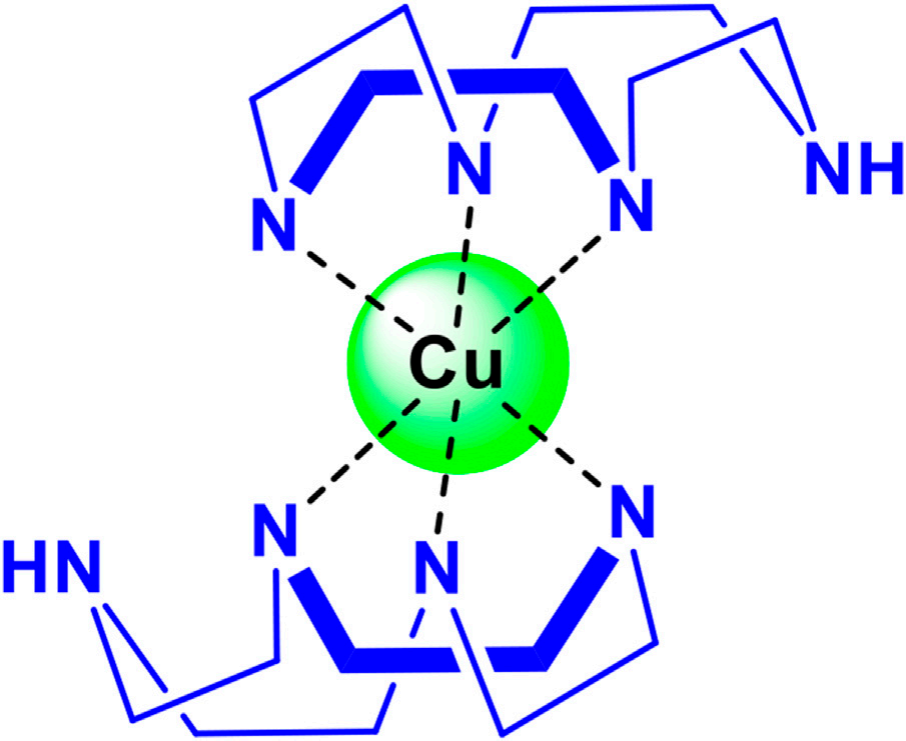
Chemical structure of Cu(II)-bis-cyclen complex.

In order to explore the catalytic properties of GO-Cu(II)-bis-cyclen nanocomposite, we have performed Cu^II^AAC alkyne–azide coupling reaction in green solvents. The plausible structure of the Cu(II)-bis-cyclen complex is depicted in Figure 1. The formation of the Cu(II)-bis-cyclen complex was confirmed by FTIR and mass-ESI spectrometry. FT-IR spectrum of Cu(II)-bis-cyclen nanocomposite (Figure 15, *refer* ESI) shows the characteristic peaks at 3,754 cm^−1^ and 3,526 cm^−1^ correspond to NH_str_ and 1,377 cm^−1^ corresponds to C-N of the secondary amine of the cyclen ligand’s stretching vibrations present in the GO-Cu(II)-bis-cyclen nanocomposite. By means of the ESI-MS technique, the formation of the molecular ion peak of Cu(II)-bis-cyclen was observed at 401.90 [*m/z*-2Cl O _4_]^+^, which infers the formation of hexaaza-coordinated, stable copper(II) complex.

**FIGURE 2:**
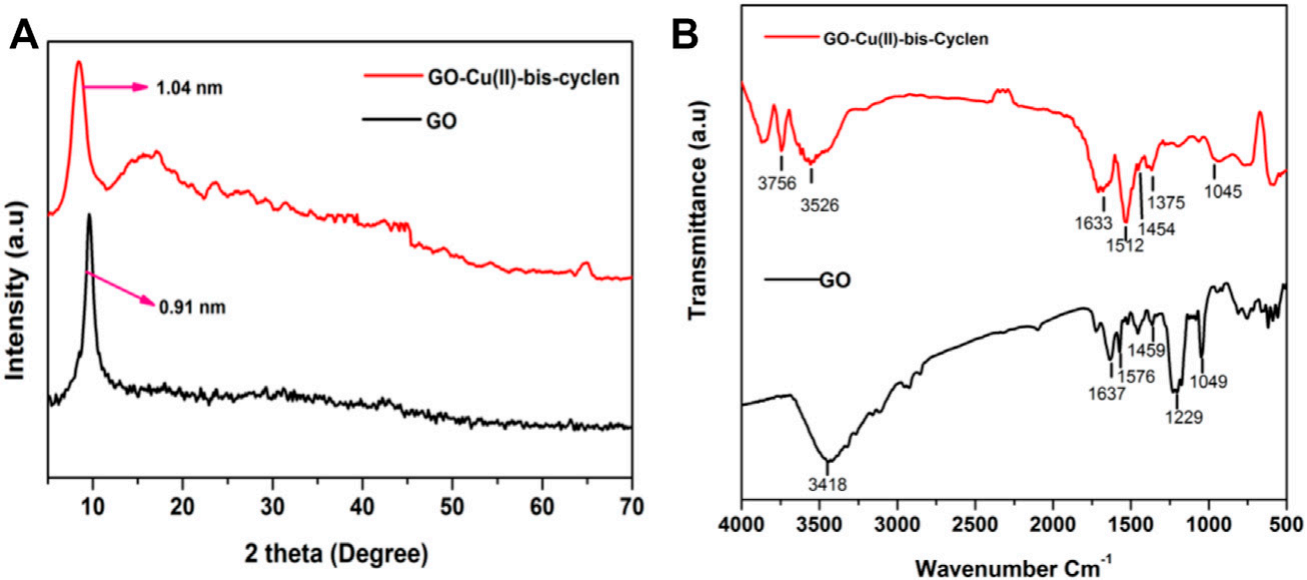
**(A)** XRD spectrum; **(B)** FT-IR spectrum.

The GO-Cu(II)-bis-cyclen nanocomposite was completely characterized by spectroscopic and microscopic techniques. As a result of intercalation of Cu(II)-bis-cyclen complex into the basal planes of GO, the powder XRD patterns of GO-Cu(II)-bis-cyclen nanomaterial show the diffraction peak at a 2θ value of 8.47° with an enhanced interlayer *d*-spacing of 1.04 nm (Figure 2A), whereas the precursor GO material displays the diffraction peak at 2θ value of 9.8° with the interlayers *d*-spacing. The intercalation of the nanocomposite was confirmed by the enhancement of the interlayer *d*-spacing. In addition, the notable diffraction peaks at 38.08° correspond to (111) plane of monoclinic CuO (JCPDS 80-1917), and at 42.00°, 48.60° were indexed to (111) and (200) planes of metallic Cu particles (JCPDS 04-0836) of the GO-Cu(II)-bis-cyclen nanocomposite (Chen et al., 2010; Tsoufis et al., 2014; Zhang et al., 2015). The FT-IR spectrum of GO-Cu(II)-bis-cyclen nanocomposite (Figure 2B) shows the characteristic peaks at 3,756 cm^−1^ and 3,526 cm^−1^ correspond to NH_str_ and −OH_str_ vibrations, respectively. The peaks at 1,633 cm^−1^ and 1,512 cm^−1^ correspond to C = O and C=C_str_ vibrations of GO-Cu(II)-bis-cyclen nanocomposite, respectively. The peak at 1,375 cm^−1^ corresponds to the C-N (amine) stretching vibrations, which was not present in the GO, and the peak at 1,045 cm^−1^ was attributed to the C-O-C_str_ vibrations of GO-Cu(II)-bis-cyclen nanocomposite.

**FIGURE 3:**
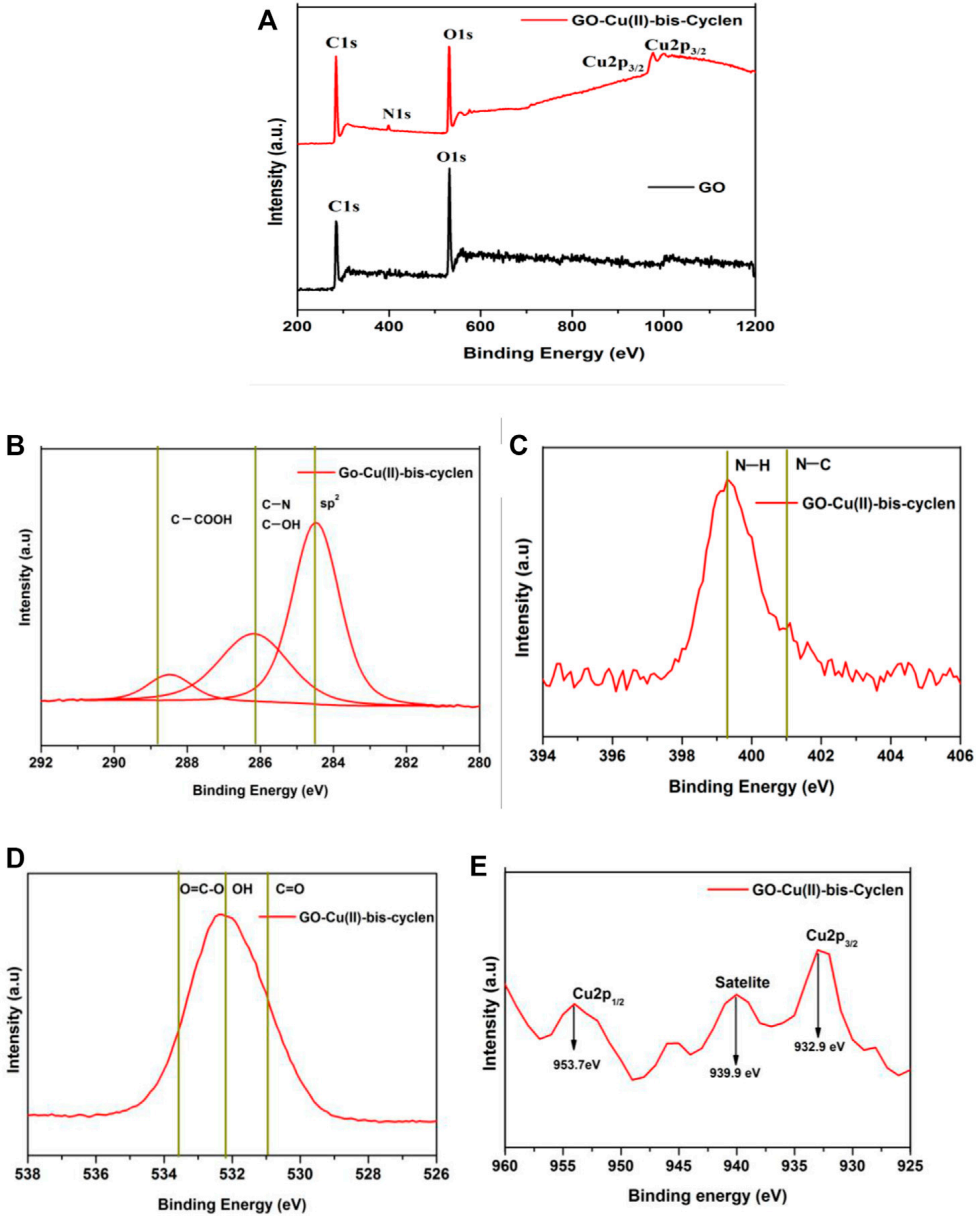
**(A)** XPS survey spectrum of GO-Cu(II)-bis-cyclen nanocomposite; **(B)** deconvoluted C1 spectrum of GO-Cu(II)-bis-cyclen nanocomposite; **(C)** deconvoluted N1s spectrum of GO-Cu(II)-bis-cyclen nanocomposite; **(D)** deconvoluted O1 spectrum of GO-Cu(II)-bis-cyclen nanocomposite; **(E)** core-level spectrum of Cu 2p of GO-Cu(II)-bis-cyclen nanocomposite.

The effect of intercalation, oxidation state of copper metal, binding energies, and the surface composition of the as-prepared nanocomposite were evaluated using the XPS analysis. The XPS survey spectrum of GO-Cu(II)-bis-cyclen nanocomposite shows the significant binding energy at 399–405 eV for N1s (Figure 3A) and at 931–952 eV for the core-level spectrum of Cu2p, which confirm the intercalation of Cu(II)-bis-cyclen complex in between the GO basal planes. The C1 spectrum of the nanocomposite shows the deconvoluted bands at 284.2, 286.5, and 288.4 eV correspond to C-C/C=C, C-OH, and COOH, respectively (Figure 3B). The N1 spectrum of GO-Cu(II)-bis-cyclen material shows the two deconvoluted bands at 399.7 and 401.7 eV were assigned to the N-H of amine and protonated N-atoms, respectively (Figure 3C). The O1 spectrum (Figure 3D) shows the two deconvoluted bands at 531 and 532.5 eV were due to the -OH of primary alcohol and C=O of carboxylic acid, respectively. Moreover, the binding energies at 953.7 and 932.9 eV correspond to the spin–orbit splitting components of Cu 2p_1/2_ and Cu 2p_3/2_ of Cu^+^ and Cu^2+^ species of GO-Cu(II)-bis-cyclen nanocomposite, respectively (Figure 3E). Further satellite peaks for Cu 2p_3/2_ and Cu 2p_1/2_ were observed at 939.9 and 962 eV that indicate the +2 oxidation state of copper metal in the GO-Cu(II)-bis-cyclen nanocomposite (Zhang et al., 2012; Saha et al., 2016; Kumar et al., 2019).

**FIGURE 4:**
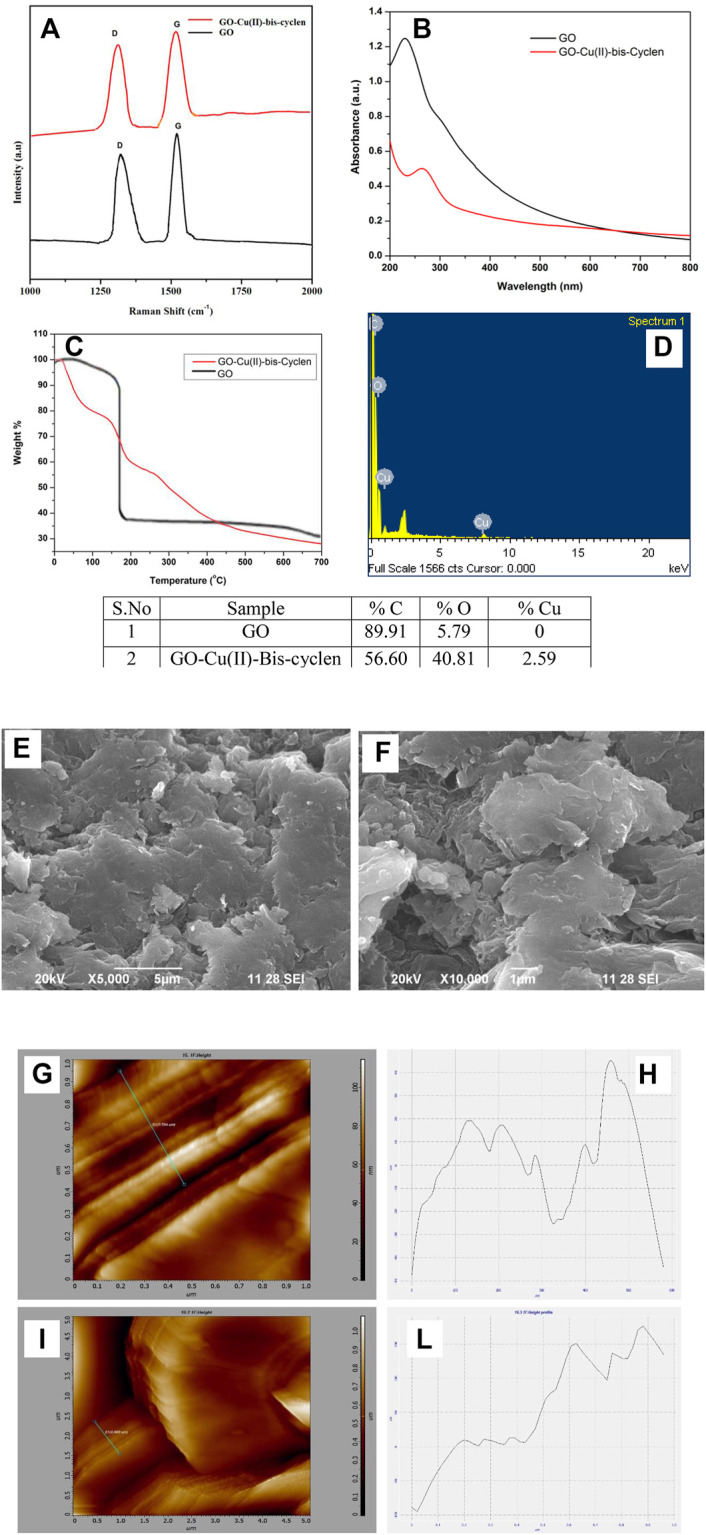
**(A)** Raman spectra of GO-Cu(II)-bis-cyclen and GO; **(B)** UV spectrum; **(C)** TGA of GO, GO-Cu(II)-bis-cyclen nanocomposite; **(D)** EDS quantification of GO-Cu(II)-bis-cyclen complex; **(E,F)** SEM images; **(G,I)** AFM images of GO-Cu(II)-bis-cyclen nanocomposite; **(H,J)** the height profile of the AFM image.

Raman spectrum was recorded to characterize the intercalated GO material by featuring the two main modes, the D- and G-modes, as the most direct and non-destructive methods. The bonding vibrations of the sp (Meldal and Schoffelen, 2016) carbon skeleton of the graphitic lattice correspond to the G-band and the defects and disorders in the structure correspond to the D-band due to the intercalation. Therefore, the increased intensity of the D band represents more defects and disorderness in the structure. The GO-Cu(II)-bis-cyclen nanocomposite shows that the two bands at 1,314.93 and 1,529.81 cm^−1^ were assigned to D-band and G-band, respectively (Figure 4A), whereas pristine GO shows the G and D bands appears at 1,592 and 1,349 cm^−1^, respectively (Ramesh and Bhagavathsingh, 2017). The I_d_/I_g_ value was found to be 0.85 for GO-Cu(II)-bis-cyclen nanocomposite (Kudin et al., 2008; Park and Yan, 2013). The UV absorbance of as-prepared nanocomposite shows the shifted peak at 264 nm was assigned to n-π* transitions of C = N bonds (Figure 4B). The redshift of the nanocomposite indicates the lone pair electrons present in nitrogen atoms of the intercalant complex interacts with GO (Bae et al., 2010). The thermal stability of intercalated GO-Cu(II)-bis-cyclen nanocomposite was investigated by TGA and is shown in Figure 4C. The nanocomposite shows an initial weight loss of around 18% at 100°C due to the removal of intercalated water molecules. The significant weight loss at 150–210°C (23%) was attributed to the thermal decomposition of oxygen-containing functionalities on the GO surface. The decomposition of the intercalated Cu(II) complex on the surface of GO was observed from the temperature at 270–700°C, which confirms the intercalation facilitates the thermal stability of GO-Cu(II)-bis-cyclen composite (Najafi and Rajabi, 2015; Gupta and Saha, 2012). The microstructure and morphology of GO sheets upon the intercalation of Cu(II)-bis-cyclen complex were investigated by SEM analysis. The SEM images of the as-prepared nanocomposite are shown in Figures 4D,E. The sheet-like structure with the wrinkled surface having granulate surface textures with spherical edges in the SEM images was due to the intercalation of Cu(II)-bis-cyclen complex on the GO plane. The EDS of GO-Cu(II)-bis-cyclen confirms no impurities, except elements such as C, N, O, and Cu (Figure 4F) (Kuila et al., 2012)

The AFM analysis of GO-Cu(II)-bis-cyclen was performed in order to relate the surface morphological features and the local electronic properties of the intercalated GO nanocomposite material. The AFM images show the curved appearance of a few-layered nanocomposite, which was due to the non-covalent interaction of the intercalant on the GO basal planes (Figures 4G–J). The height profiles of the AFM images show the few layer thickness ranges from 8 to 35 nm with the uniform layered arrangement.

### Cyclic Voltammetry Studies

**FIGURE 5:**
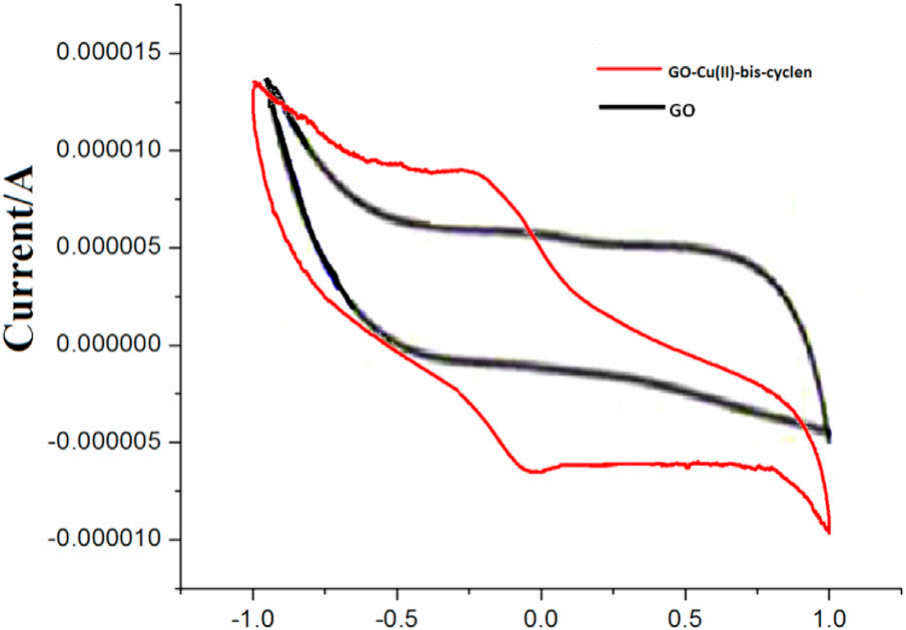
Cyclic voltammogram of GO-Cu(II)-bis-cyclen nanocomposite and GO nanosheet.

The CV curves of GO and GO-Cu(II)-bis-cyclen catalyst are in rectangular shapes of voltammograms and are symmetric in anodic and cationic directions which proves that there is a difference in the rate of the electron transfer process (Figure 5). GO-Cu(II)-bis-cyclen catalyst confirms the one-electron redox process from the CV curve, which is due to the reduction of Cu^II^ to Cu^I^ species supporting the first step of the click catalytic cycle, that is, reduction of Cu(II) species to Cu(I) species occurs on the addition of the reductant after which the reduced species attacks acetylene to form copper acetylide intermediate, thereby facilitating the consecutive cycloaddition mechanism. Additionally, this class of GO-Cu^II^-bis-cyclen nanocomposite was considered to be the quasi-reversible system as the observed potential difference of anodic and cathodic peak potential of GO-Cu^II^-bis-cyclen nanocomposite was found to be greater than 59 mV (Table 1). Considering the redox potential of the nanocomposite shows the significant intercalation of the respective complex onto the GO-planes. Thus, the Cu^II^/Cu^I^ redox potential of the GO-Cu(II)-bis-cyclen nanocomposite was proved to be an efficient catalyst in stabilizing Cu(I) species in accelerating catalysis to yield 1,4-disubstituted-1,2,3-triazoles.

**TABLE 1:** Voltammetric values of the reduction potentials E_c_, ∆E and E_1/2_.

S.No	GO catalyst	Epc (V)	Epa (V)	Δe (V)	E1/2 (V)
1	GO-Cu(II)-bis-cyclen nanocomposite	−0.107	−0.205	−0.098	−0.156

### Click Reactions Using GO-Cu(II)-Bis-Cyclen Nanocomposite

In this study, the as-prepared Cu(II)-bis-cyclen complex was intercalated onto the GO basal planes and the heterogeneous nanocomposite was isolated in order to test its performance as the click catalyst for the cycloaddition of alkynes and alkynes (CuAAC) reaction in green solvents. The organic azide precursors were synthesized by the nucleophilic substitution reaction of sodium azide with the various organic halides for the isolation of corresponding triazoles as a product of click reaction (Wang et al., 2001). Typically, the azides (1b-7b) were synthesized by the reaction of the respective organic halides with sodium azide in the biphasic mixture of green solvents at 0°C to RT. The addition of the substantiate quantity of water offers the performance of the reactants in safer experimental conditions to prevent the potential explosion of azidation.

**SCHEME 1:**
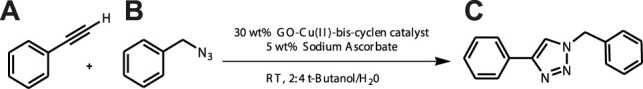
Optimization of the synthesis of triazole using GO-Cu (II)-bis-cyclen catalyst.

The classic click reaction of phenyl acetylene with benzyl azide was performed using the as-prepared GO-Cu(II)-bis-cyclen (30 wt%) as a click catalyst and sodium ascorbate (5 wt%) as a reductant in *t*-butanol/H_2_O as a green solvent mixture at RT. The desired product, 1,4-disubstituted-1,2,3 triazole, was isolated as 89% yield using the developed nanocomposite catalyst (Scheme 1).

The effect of GO-Cu (II)-bis-cyclen catalyst in the presence and absence of sodium ascorbate was also studied in order to optimize the CuAAC reaction. The 30 wt% loading of click catalyst yields the corresponding 1,2,3-triazole in good yield (up to 89%) when the cycloaddition reaction was performed between phenyl acetylene and benzyl azide at room temperature in the presence of the reductant.

### Reusability of the Intercalated Nanocomposite

The efficiency of the developed heterogeneous catalyst was tested with the same condition after several times of washing. After the first cycle completion of the click reaction, the heterogeneous catalyst GO-Cu(II)-bis-cyclen nanocomposite was filtered and washed with water (5 ml) and ethanol thrice (5 ml), followed by diethyl ether to get a free flow solid. The solid GO-Cu(II)-bis-cyclen nanocomposite catalyst was dried at room temperature for 12 h and used for the subsequent batches of click reaction using the substrates phenyl acetylene and benzyl azide to isolate the triazole product in moderate yield (Table 2).

**TABLE 2:** Recyclability of GO-C1 nanocomposite materials and their yield.

Entry	GO-Cu(II)-bis-cyclen nanocomposite^a^	Yield (%)^b^
1	Click reaction	89
2	Recycle 1	72
3	Recycle 2	60
4	Recycle 3	51

a Reaction condition: phenyl acetylene (1 mmol), benzyl azide (1 mmol) GO-Cu(II)-bis-cyclen catalyst (30 wt%), and sodium ascorbate (5 wt%) were reacted in *t*-butanol/H_2_O (2:4 *v/v*) at RT.

b Isolated yield of 1-benzyl-4-phenyl-1*H*-1,2,3-triazole.

### Optimized Conditions of Click Reaction Catalyzed by GO-Cu(II)-Bis-Cyclen Nanocomposite

The effect of catalyst dosage and solvent was studied by optimizing the reaction conditions with the click substrates of phenyl acetylene and benzyl azide using the GO-Cu(II)-bis-cyclen catalyst. From Table 3, it was inferred that 30 (mol%) of GO-Cu(II)-bis-cyclen was the most effective catalytic amount for the click reaction and the higher yield for the isolation of triazole compounds in the solvent mixture *t*-butanol/H_2_O (2:4 *v/v*).

**TABLE 3:** List of the azides utilized, reaction time, the triazoles isolated, and the isolated yield.

Entry	R-N_3_ (1–7b)	Time (mins)	Isolated product (1c-7c)	Yield (%)
1	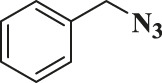	60	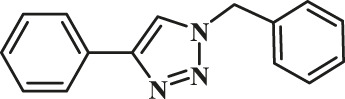	89
2	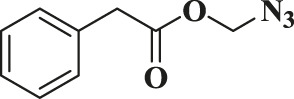	70	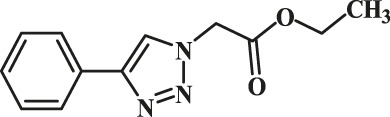	86
3	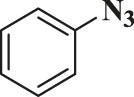	65	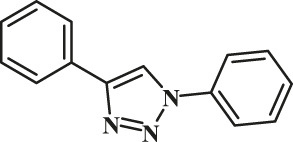	83
4	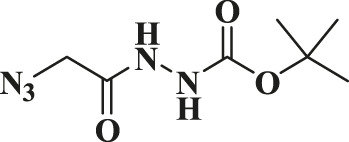	60	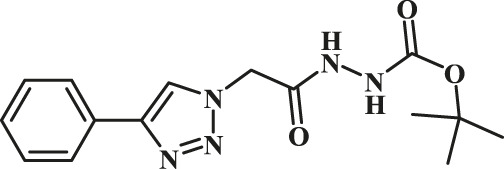	85
5	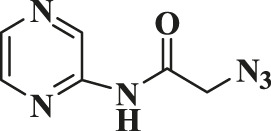	72	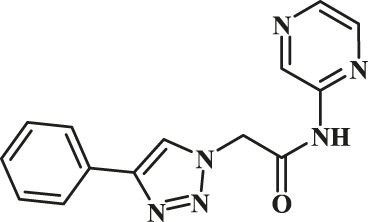	72
**6**	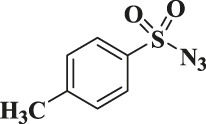	75	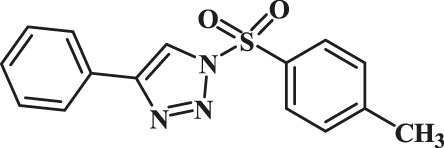	69
**7**	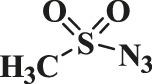	80	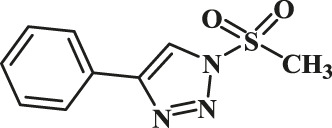	64

To demonstrate the scope of the CuAAC reaction using GO-Cu(II)-bis-cyclen catalyst, the series of azides containing an electron-donating and an electron-withdrawing group were adapted to perform the click reaction with phenyl acetylene under optimized conditions for the isolation of 1,4-disubstituted-1,2,3-triazoles, and the results obtained are given in Table 4. It was observed that the performance of the Cu(II)-bis-cyclen–intercalated GO nanocomposite was convenient, reproducible, and reusable as a click catalyst for alkyl and aryl azide substrates.

**TABLE 4:** Various solvent effects for the isolation of better yields.

Entry	GO-Cu(II)-bis-cyclen catalyst (mol%)^a^	Solvent	Yield (%)^b^
1	—	*t*-butanol/H_2_O (2:4 *v/v*)	0
2	10^c^	*t*-butanol/H_2_O (2:4 *v/v*)	0
3	10	*t*-butanol/H_2_O (2:3 *v/v*)	43
4	10	*t*-butanol/H_2_O (2:4 *v/v*)	50
5	15	*t*-butanol/H_2_O (2:4 *v/v*)	76
6	20	*t*-butanol/H_2_O (2:4 *v/v*)	81
7	30	*t*-butanol/H_2_O (2:4 *v/v*)	89
8	30	*t*-butanol	67
9	30	Ethanol	77
10	30	CH_3_CN	64
11	30	H_2_O	78
12	30	DMSO	51

a Reaction conditions: phenyl acetylene (1 mmol), benzyl azide (1 mmol), and sodium ascorbate (5 mg) were reacted at RT.

b Isolated yield of 1-benzyl-4-phenyl-1*H*-1,2,3-triazole.

c Without the catalyst, sodium ascorbate.

While examining the mechanistic pathway of CuAAC in the presence of heterogeneous GO-Cu(II)-bis-cyclen catalyst, the “Breslow effect” and hydrophilic GO support have promoted the high catalytic activity of GO-Cu(II)-bis-cyclen nanocomposite (Breslow, 2004; Putta et al., 2015). Thus, the highlight of the versatile characteristic of the GO-Cu(II)-bis-cyclen nanocomposite is ascribed to the excellent dispersity of the catalyst in water, hydrophilic nature of the GO for the accumulation of organic substrates in water, and in the “Breslow effect.” According to the “Breslow effect,” the reaction in the organic/aqueous solvent mixture, the functionalized GO catalyst (GO-Cu(II)-bis-cyclen), disperses well in the organic/aqueous solvent mixture, which eventually promotes the cycloaddition of phenyl acetylene and aryl azide in increasing the yields of 1,2,3-triazoles. In order to support the “Breslow effect” with the experimental data during the optimization study of the reaction conditions with the click substrates of phenyl acetylene and benzyl azide using the GO-Cu(II)-bis-cyclen catalyst, the solvent effect was demonstrated. It is inferred that the triazole product was isolated in a higher yield (89%) with a catalyst loading of 30 mol% of GO-Cu(II)-bis-cyclen nanocomposite in the solvent mixture *t*-Butanol/H_2_O (2:4 *v/v*). We have examined some control reaction without water as a cosolvent in order to validate the aforementioned conclusions; however, the yields are less than the reactions performed with *t*-butanol/H_2_O (2:4 *v/v*).

**SCHEME 2:**
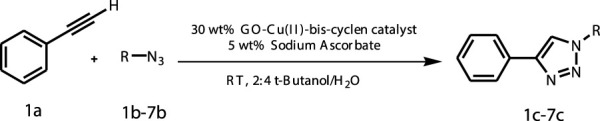
General schematic representation of the Cu^II^AAC reaction using GO-Cu(II)-bis-cyclen catalyst.

During the cycloaddition reaction, the addition of the catalytic amount of the sodium ascorbate conveniently facilitates the reduction of the Cu(II)-bis-cyclen complex decorated on the GO surface, which in turn promotes the stabilization of *in situ*–formed Cu(I) species by accelerating the rapid electron transfer process. The heterogeneous GO-Cu(II)-bis-cyclen catalyst non-covalently supported on the GO accelerates the CuAAC reaction for the formation of the respective triazoles in higher yields (Scheme 2).

## Conclusion

This class of heterogeneous, reusable Cu(II)-bis-cyclen complex decorated on the graphene oxide basal planes was successfully synthesized by the facile, non-covalent intercalation and proved to be an efficient catalyst for the CuAAC reaction to isolate 1,4-disubstituted-1,2,3-triazoles in good yield (89%). The XRD patterns of the nanocomposite show the enhanced interlayer *d*-spacing of 1.04 nm due to the intercalation of the Cu(II)-bis-cyclen complex. The XPS spectrum was also confirmed by the nanocomposite material with the significant binding energies of 399–405 eV for N1s and 932.3 eV for Cu, which was evident of intercalation onto the GO surface. The morphology of the nanocomposite was visualized by the microscopic images of SEM and AFM. The successful utilization of the as-prepared heterogeneous copper-based GO nanocomposite as a click catalyst for the copper mediated azide–alkyne cycloaddition with various organic azide substrates was achieved. The heterogeneous intercalated GO click catalyst could be useful for the conjugation of biomacromolecules through a triazole moiety in the green solvent system.

## Data Availability

The original contributions presented in the study are included in the article/Supplementary Material; further inquiries can be directed to the corresponding author.
