# Supplementary material for: Copper(II)-Bis-Cyclen Intercalated Graphene Oxide as an Efficient Two-Dimensional Nanocomposite Material for Copper-Catalyzed Azide–Alkyne Cycloaddition Reaction
Source: Front Chem. 2022 Jan 7;9:754734. doi: 10.3389/fchem.2021.754734 (PMC8782203; doi:10.3389/fchem.2021.754734)
Supplement: Supplementary file 1 [file DataSheet1.docx]

**Copper(II)-Bis-Cyclen Intercalated Graphene Oxide as an Efficient Two Dimensional Nanocomposite Material for Copper-Catalyzed Azide-Alkyne Cycloaddition Reaction**

Angel Green Samuel,^1^ S. Sowmya, ^1^ Vijendran Vijaikanth,^1^ Bhagavathsingh Jebasingh^1*^

^1^Department of Applied Chemistry, Karunya Institute of Technology and Sciences, Coimbatore – 641114, Tamilnadu, INDIA.

^*^[jebasinghb@karunya.edu](mailto:jebasinghb@karunya.edu)

**Supporting Information**

**Table of Contents**

| **S.No** | **Contents** | **Page No** |
| --- | --- | --- |
| 1 | Spectroscopic data of the click products (1c-7c) | S1 |
| 2 | Spectral Characterizations of the triazole products | S4 |
| 3 | FT-IR and Mass spectra of Cu(II)-bis-cyclen- complex | S18 |
| 4 | References | S19 |

**1. Spectroscopic data of the click products (1c-7c)**

**1) Compound 1c**

**
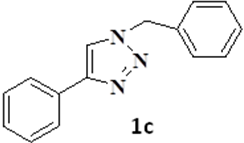
**

**1-benzyl-4-phenyl-1*H*-1,2,3-triazole (1c)**^1^ was isolated as a white solid (yield 89 %). mp: 129^°^C. ^1^H NMR (400 MHz, CDCl_3_): *δ*_H_ 5.60 (s, 2H), 7.28-7.32 (m, 3H), 7.34-7.42 (m, 5H), 7.68 (s, 1H) 7.83 (t, *J*=7.2 Hz, 2H). ^13^C NMR (100 MHz, CDCl_3_): *δ*_C_ 147.18, 133.65, 129.49, 128.12, 127.77, 127.74, 127.13, 126.71 124.66, 118.49, 53.18. ESI-MS, *m/z*: calcd. for C_15_H_13_N_3_ [M+H]^+^: 236.11, found: 236.10. IR (KBr): 3450, 1641, 698 cm^-1^.

2) **Compound 2c**

**
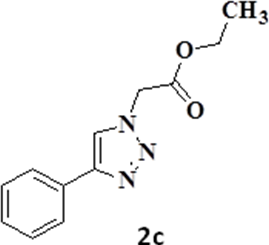
**

**(4-Phenyl-[1,2,3]triazol-1-yl)-acetic acid ethyl ester (2c)**^2,3^ was isolated as a white solid (yield 86%.) mp : 75°C. ^1^H NMR (400 MHz, CDCl_3_): *δ*_H_ 1.34 (t*, J* =7.2 Hz, 3H), 4.26 (q, *J* =7.2 Hz, 2H), 5.20 (s, 2H), 7.38-7.34 (tt, *J*=8 Hz, *J*= 1.20 Hz, 1H), 7.43-7.49 (m, 2H), 7.85-7.87 (m, 2H), 7.94 (s, 1H): ^13^C NMR (100 MHz, CDCl_3_): δ_C_ 166.31, 148.25, 130.38, 128.86, 128.29, 125.82, 121.01, 62.48, 50.97, 14.09 ESI-MS, *m/z*: calcd. for C_12_H_13_N_3_O_2_ [M+H]^+^: 232.10, found: 232.10. IR (KBr): 3448, 1635, 918 cm^-1^.

**3) Compound 3c**

**3c**

**1,4-diphenyl-1*H*-1,2,3-triazole (5c)**^2^ was isolated as a pale red solid (yield 83%). mp: 184^o^C. ^1^H NMR (400 MHz, CDCl_3_): *δ*_H_ 7.90 (s, 1H), 7.47 (m, 2H), 7.34 (m, 2H), 7.32 (m, 6H). ^13^C NMR (100 MHz, CDCl_3_): *δ*_C_ 148.20 136.93, 130.18, 129.72, 128.71, 125.72. ESI-MS, *m/z*: calcd. for C_14_H_11_N_3_ [M+H]^+^: 222.10, found: 222.10. IR (KBr): 3448, 1637, 1506, 758 cm^-1^.

4) **Compound 4c**

**4c**

**{2-[2-(4-Phenyl-[1,2,3]triazol-1-yl)-acetylamino]-ethyl}-carbamic acid tert-butyl ester (4c)** was isolated as off white solid (yield 85%).mp:152^°^C ^1^H-NMR (400MHz, DMSO): ): *δ*_H_ 1.38(s, 9H ), 3.24 ( t, *J*= 7.12 Hz, 2H), 3.41 (t, *J*= 7.12 Hz, 2H), 5.12(s, 2H), 7.32-7.35 (tt, *J*= 7.2 Hz, *J*= 1.40 Hz, 1H), 7.40-7.47 (m, 2H), 7.85 (m, 2H), 8.52(s, 1H). ^13^C NMR (100 MHz, CDCl_3_): *δ*_C_ 165.16, 147.22, 133.49, 129.26, 127.81, 127.27, 124.78, 120.02, 67.56, 49.87, 46.21, 43.27, 27.20. ESI-MS, *m/z*: calcd. For C_17_H_23_N_5_O_3_ [M+Na]^+^: 368.17, found: 368.10. IR (KBr): 3446, 1639, 1541, 1369, 769 cm^-1^

**5)Compound 5c**

**5c**

**2-(4-Phenyl-[1,2,3]triazol-1-yl)-N-pyrazin-2-yl-acetamide (5c)** was a isolated as dark yellow solid (yield 72%).mp: 181^°^C ^1^H NMR (400MHz, DMSO): ): *δ*_H_ 5.60(s, 2H), 7.20 ( tt, *J*= 8 Hz, *J*= 1.5 Hz, 1H), 7.34 (m, 2H), 7.42 (m, 2H), 7.96 (s, 1H) 8.50 (dd, *J*= 6.8 Hz, *J*= 0.50 Hz, 1H), 8.54 (dd, *J*= 6.8 Hz, *J*= 1.7 Hz, 1H), 8.68 (dd, *J*= 1.6 Hz, *J*= 0.6 Hz, 1H): ^13^C NMR (100 MHz, CDCl_3_): *δ*_C_ 170.70, 147.94, 145.58, 141.46, 135.85, 134.75, 134.17, 133.13, 132.07, 130.19, 128.26, 57.38. ESI-MS, *m/z*: calcd. For C_14_H_12_N_6_O [M+H]^+^: 281.11, found: 281.20. IR (KBr): 3477, 1695, 1560, 1421, 690 cm^-1^.

6) **Compound 6c**

**6c**

**4-Phenyl-1-(toluene-4-sulfonyl)-*1H*-[1,2,3]triazole(6c)**^4^ was isolated as a off-white solid (yield 69%). mp: 89 °C. ^1^H NMR (400 MHz, DMSO): *δ*_H_ 2.44 (s, 3H), 7.32(m, 2H), 7.38-7.34(m, 3H), 7.48-7.47(m, 2H), 7.82-7.88(m, 2H), 8.18 (s, 1H). ^13^C NMR (100 MHz, DMSO): δC 143.18, 139.11, 135.91, 130.84, 130.65, 129.49, 128.71 127.16, 126.49, 25.19. ESI-MS, *m/z*: calcd. for C_15_H_13_N_3_O_2_S [M+Na]^+^: 322.06, found: 322.00. IR (KBr): 3471, 2961, 2065, 1637, 1048 cm^-1^

**7) Compound 7c**

**7c**

1-Methanesulfonyl-4-phenyl-*1H*-[1,2,3]triazole (12c)^4,5^ was isolated as a white solid (yield 64%). mp:89 °C. ^1^H NMR (400 MHz, CDCl_3_): *δ*_H_ 3.29(s, 3H), 7.38-7.40(m, 3H), 7.41-7.45(m, 2H), 8.10(s, 1H). ^13^C NMR (100 MHz, CDCl_3_): *δ*_C_ 132.10, 130.10, 129.01, 128.21, 126.15, 41.49. ESI-MS, *m/z*: calcd. for C_9_H_9_N_3_O_2_S [M+Na]^+^: 246.03, found: 246.00. IR (KBr): 3366, 1696, 1370, 868 cm^-1^

**2. SPECTRAL CHARACTERIZATION RESULTS OF CLICK PRODUCT**

**2.1 Benzyl-4-phenyl-1H-[1,2,3]triazole (1c)**

**
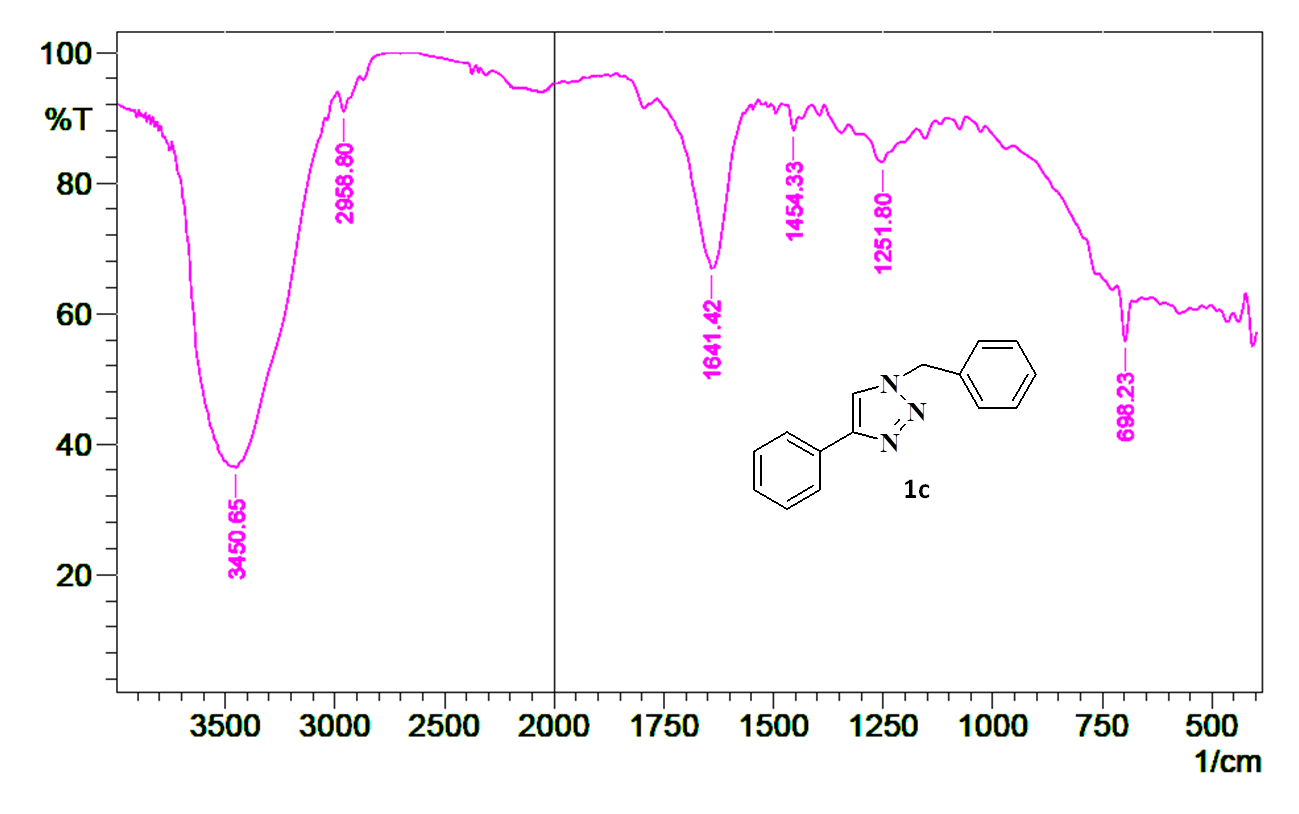
**

**
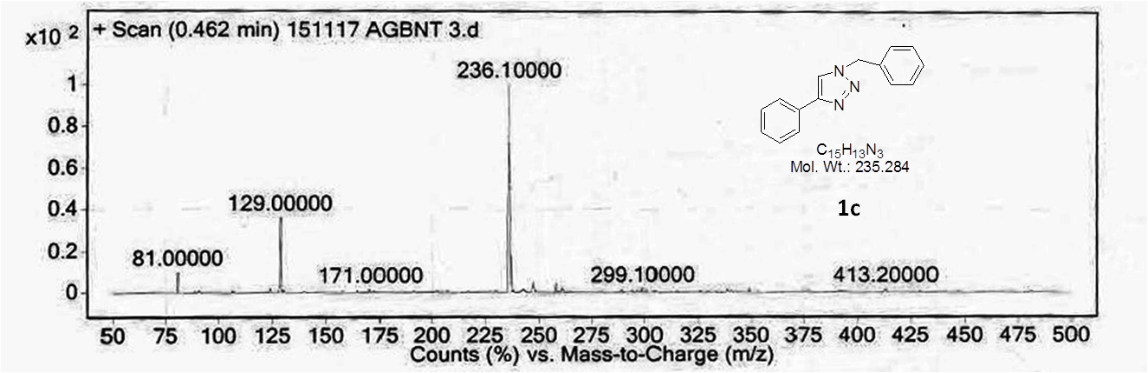
**

**Figure 1. FTIR & Mass spectrum of 1-Benzyl-4-phenyl-*1H*-[1,2,3]triazole (1c)**

**
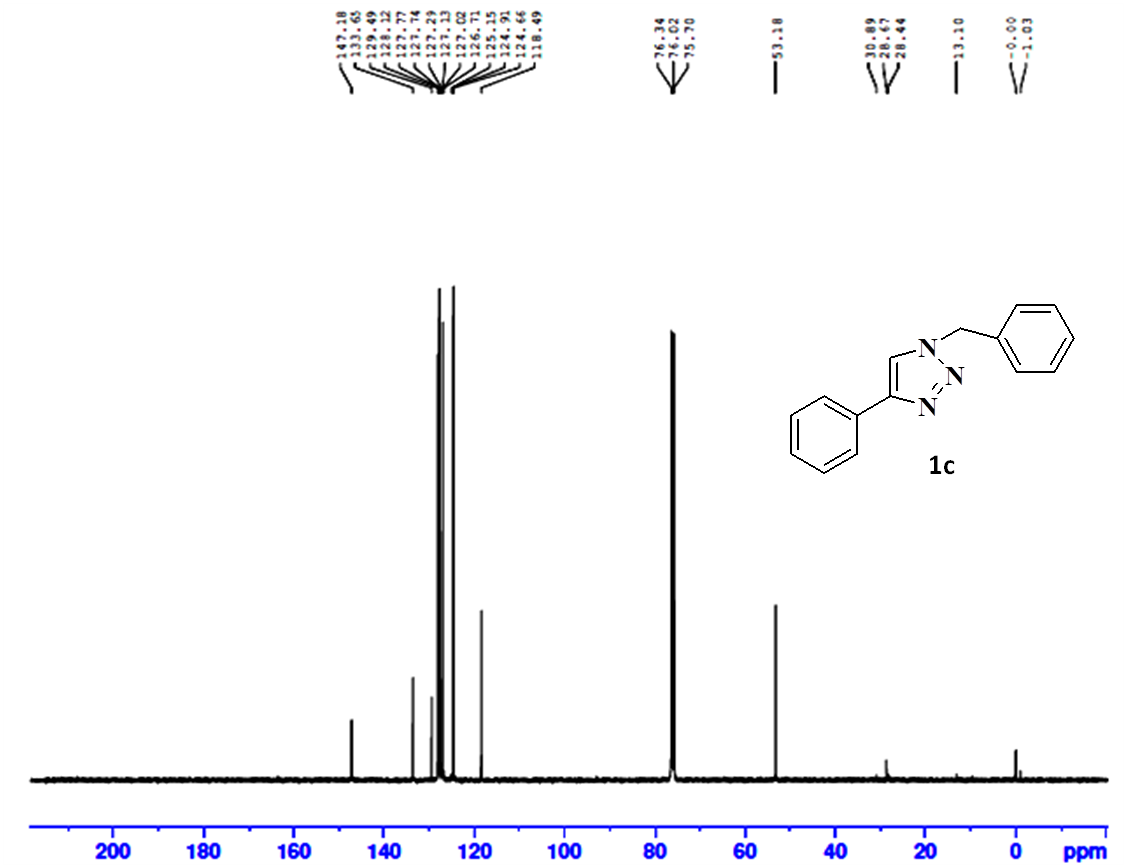
**

**
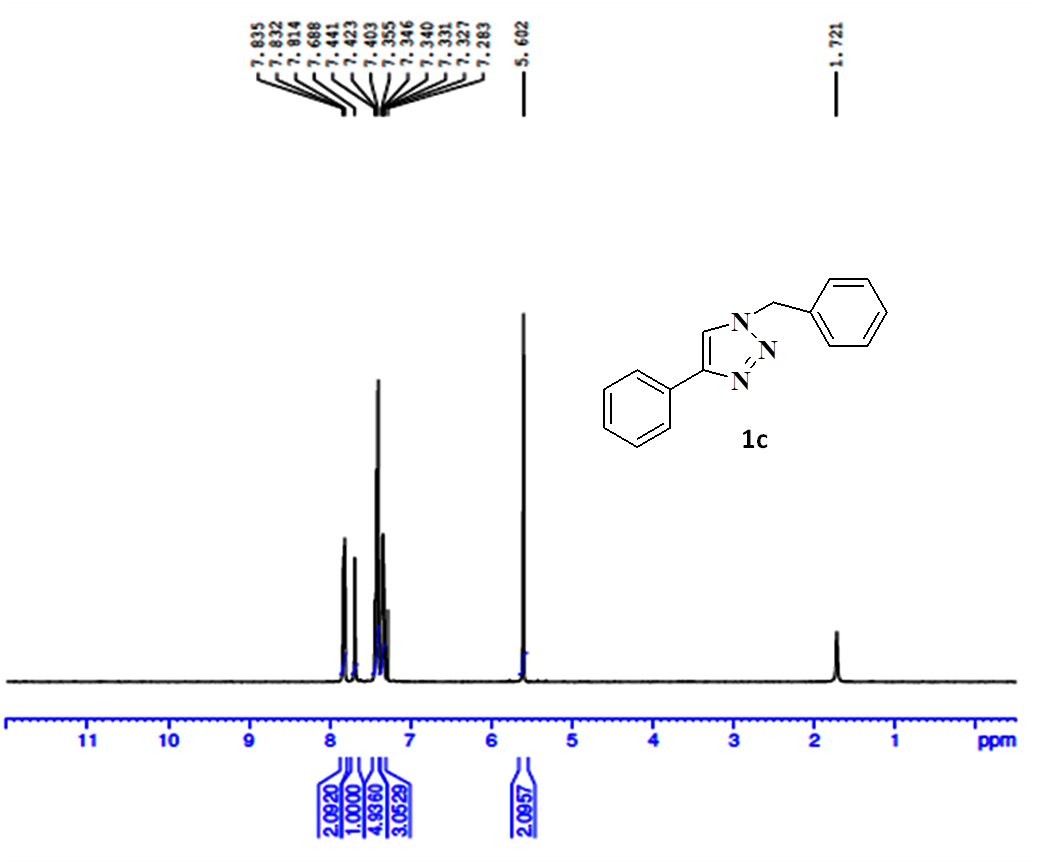
**

**Figure 2. ^1^H-NMR & ^13^C-NMR spectrum of 1-Benzyl-4-phenyl-*1H*-[1,2,3]triazole (1c)**

**2.2 (4-Phenyl-[1,2,3]triazol-1-yl)-acetic acid ethyl ester (2c)**

**
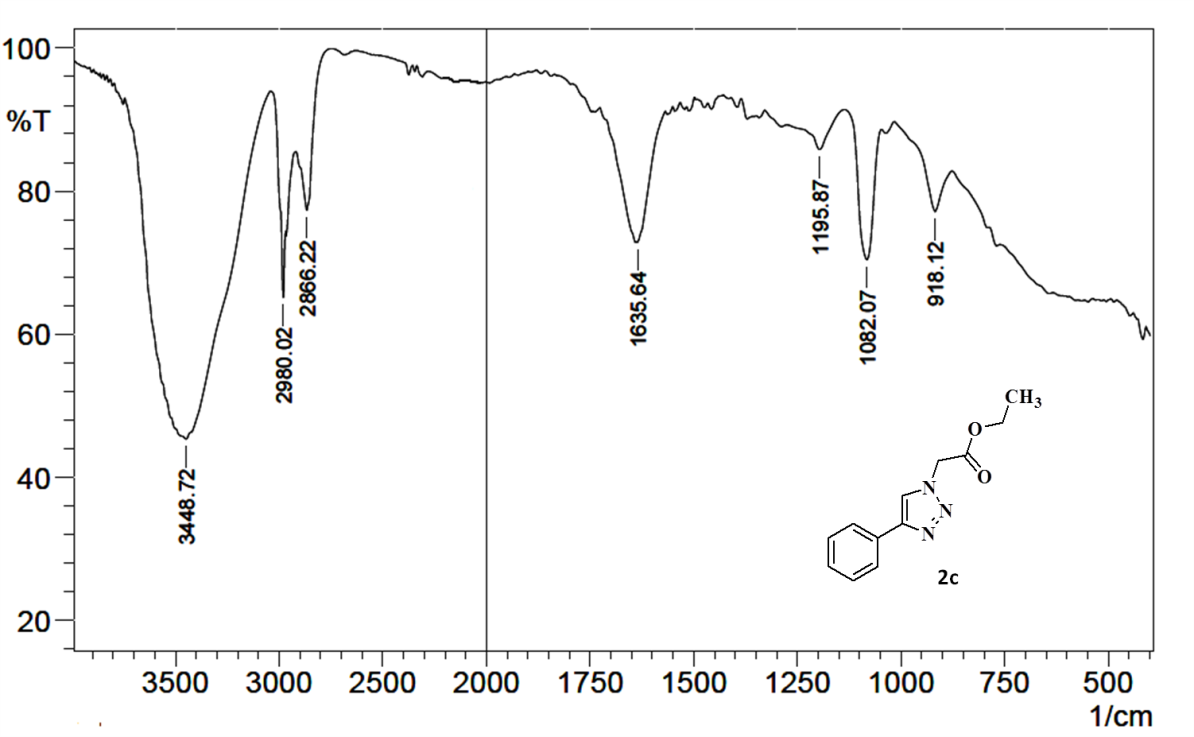
**

**
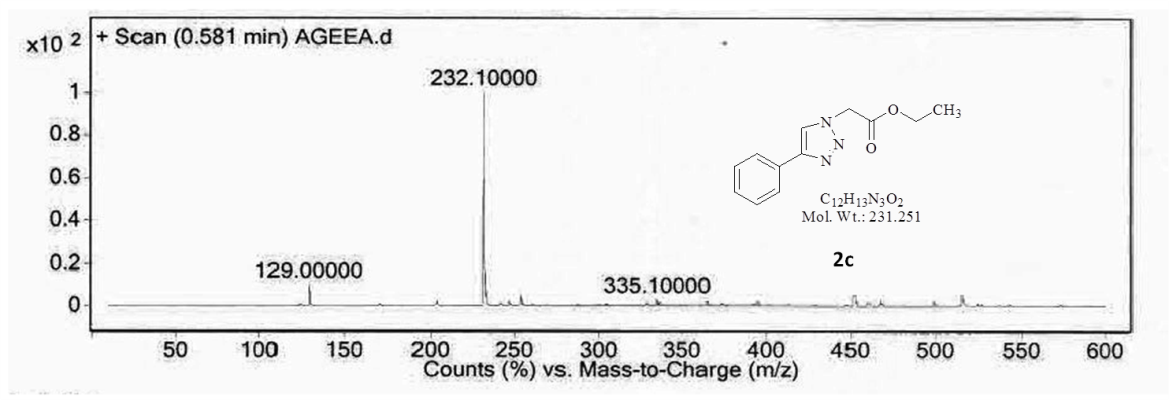
**

**Figure 3 FTIR & Mass Spectrum of (4-Phenyl-[1,2,3]triazol-1-yl)-acetic acid ethyl ester (2c)**

**
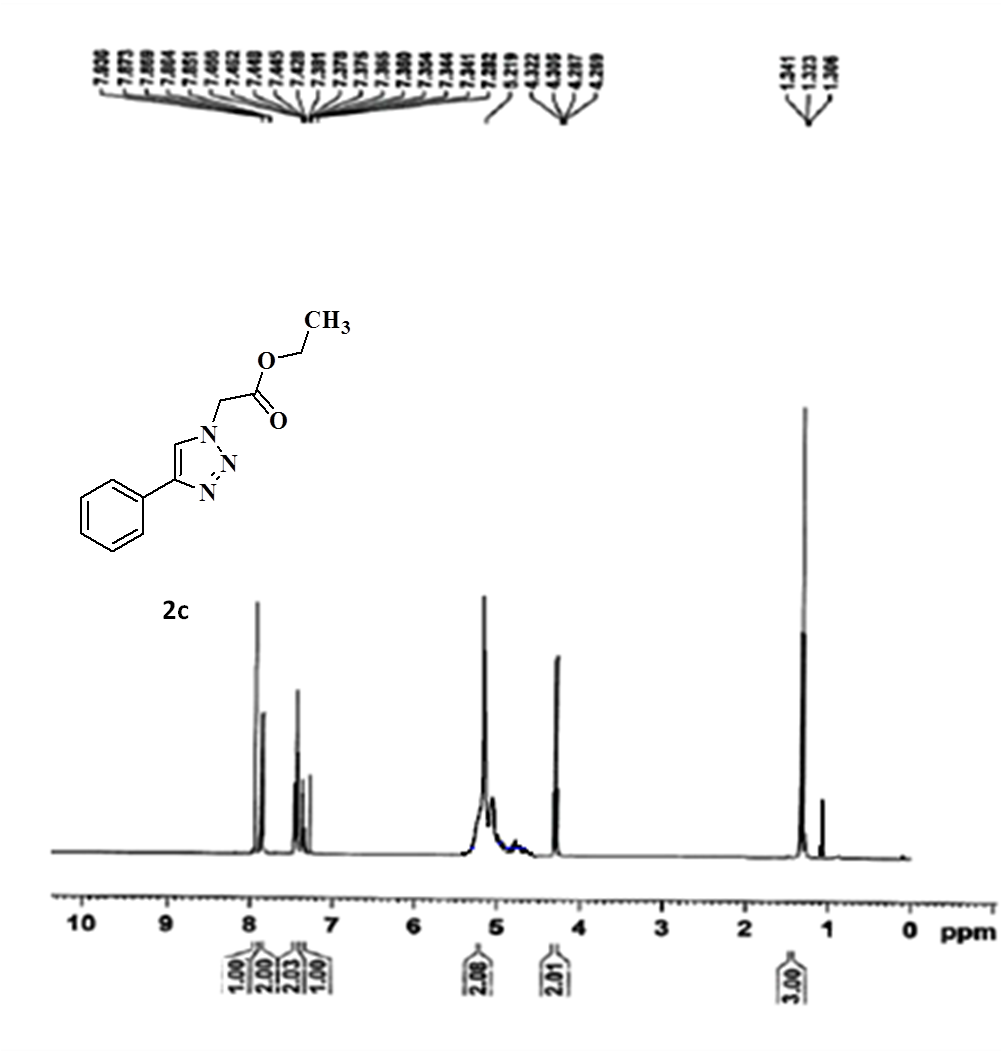
**

**
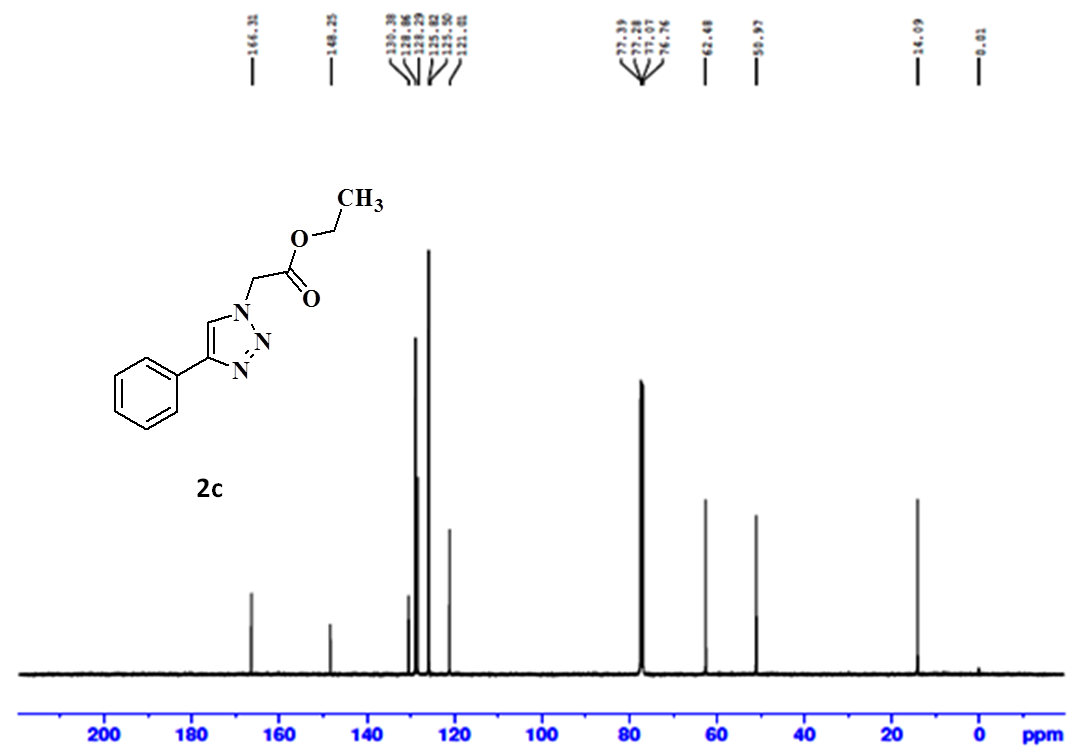
**

**Figure 4: ^1^H-NMR & ^13^C-NMR spectrum of (4-Phenyl-[1,2,3]triazol-1-yl)-acetic acid ethyl ester (2c)**

**2.3 1,4-Diphenyl-1H-[1,2,3]triazole (3c)**

**
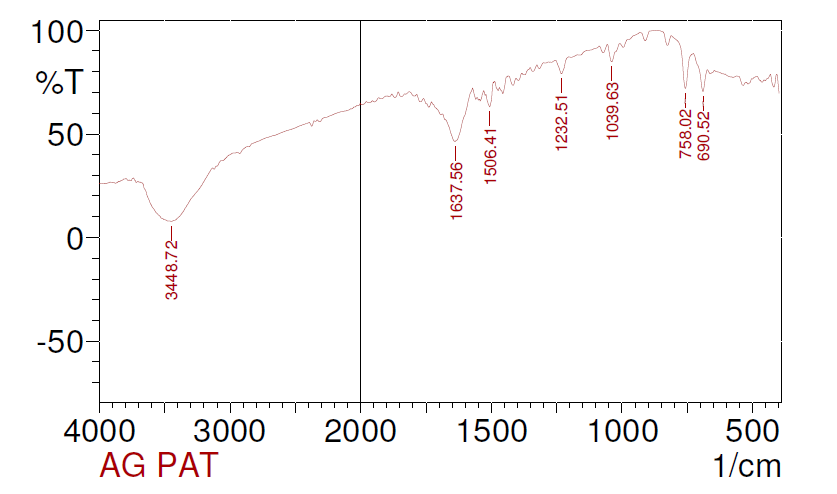
**

**3c**

**
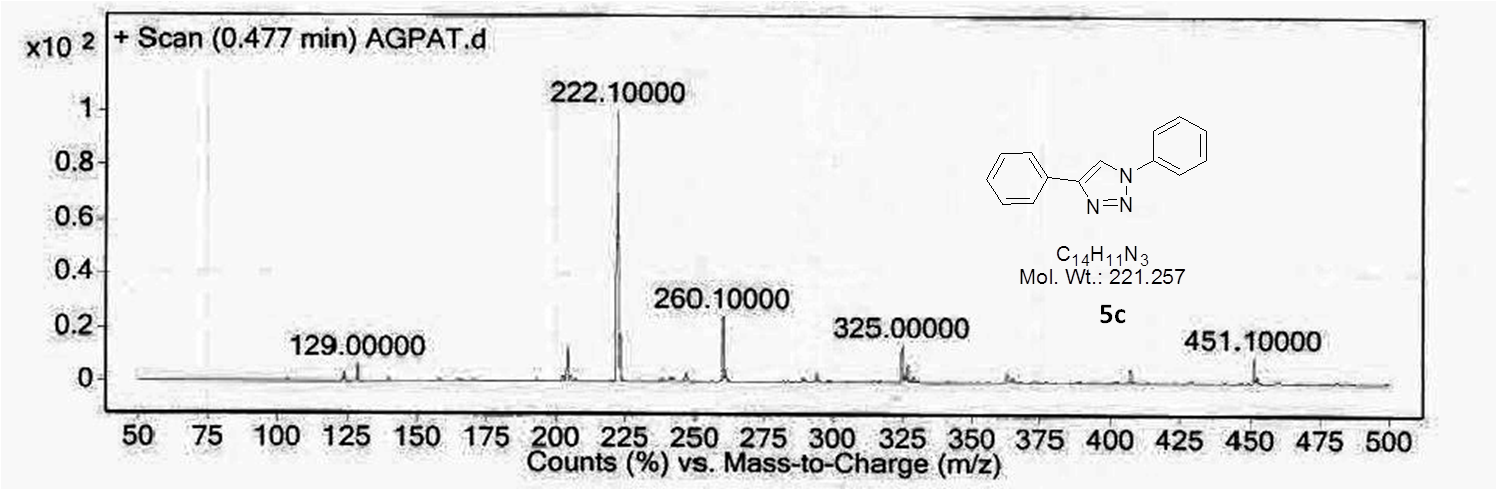
**

**3c**

**Figure 5. FTIR & Mass Spectrum of 1,4-Diphenyl-1H-[1,2,3]triazole (5c)**

**
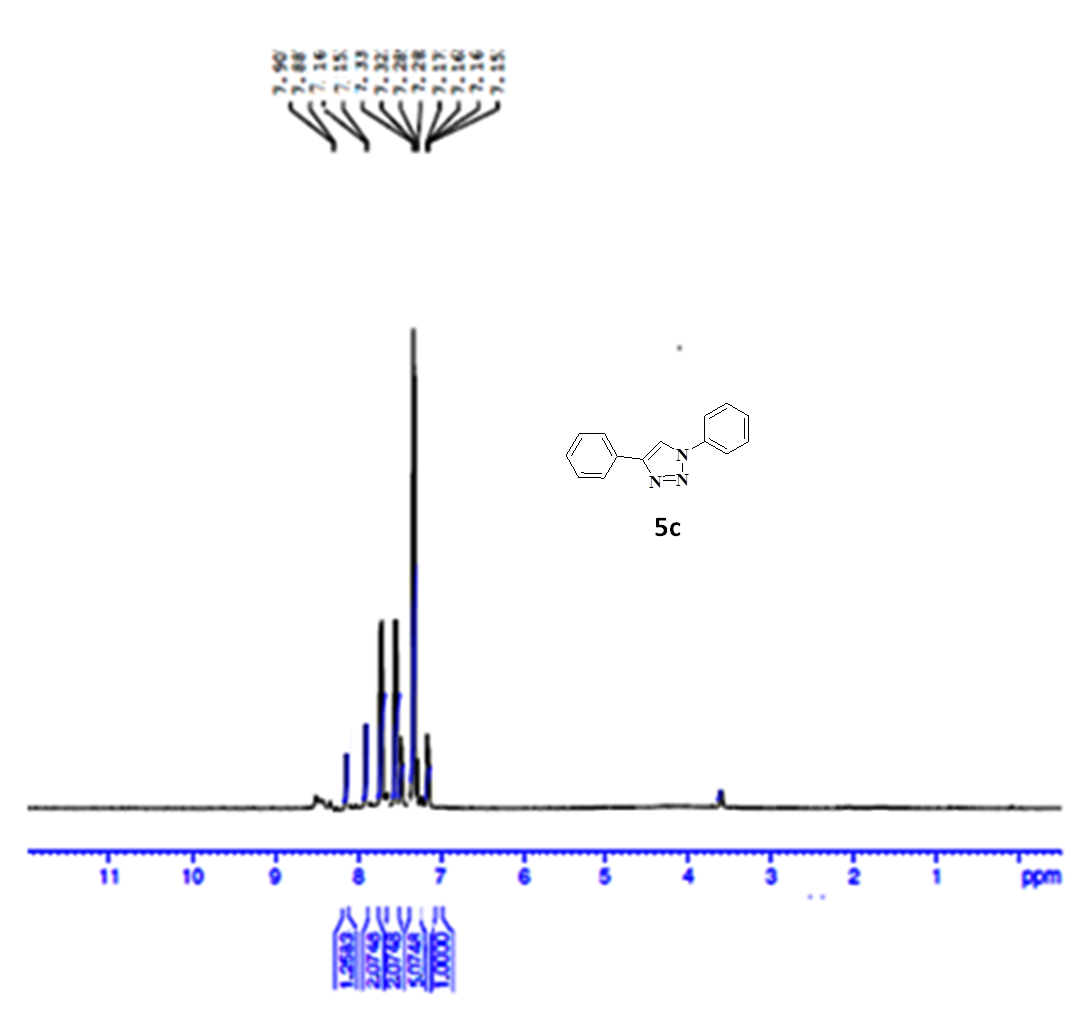
**

**3c**

**
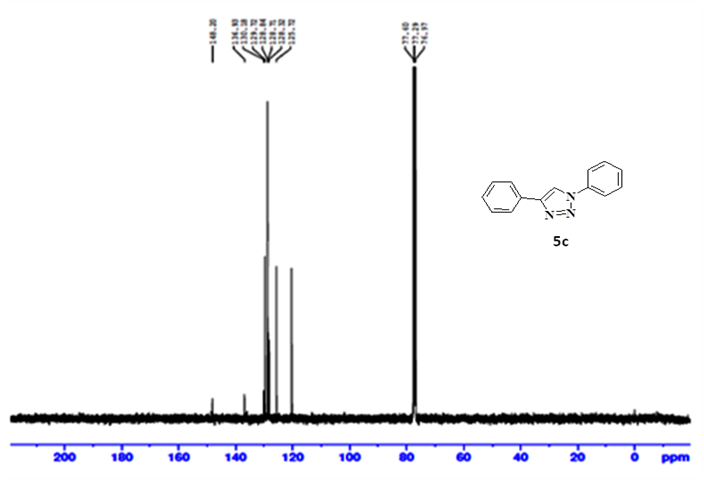
**

**3c**

**Figure 6.^1^H-NMR & ^13^C-NMR Spectrum of 1,4-Diphenyl-1H-[1,2,3]triazole (3c)**

**2.4 N'-[2-(4-Phenyl-[1,2,3]triazol-1-yl)-acetyl]-hydrazinecarboxylic acid tert-butyl ester (6c)**

**
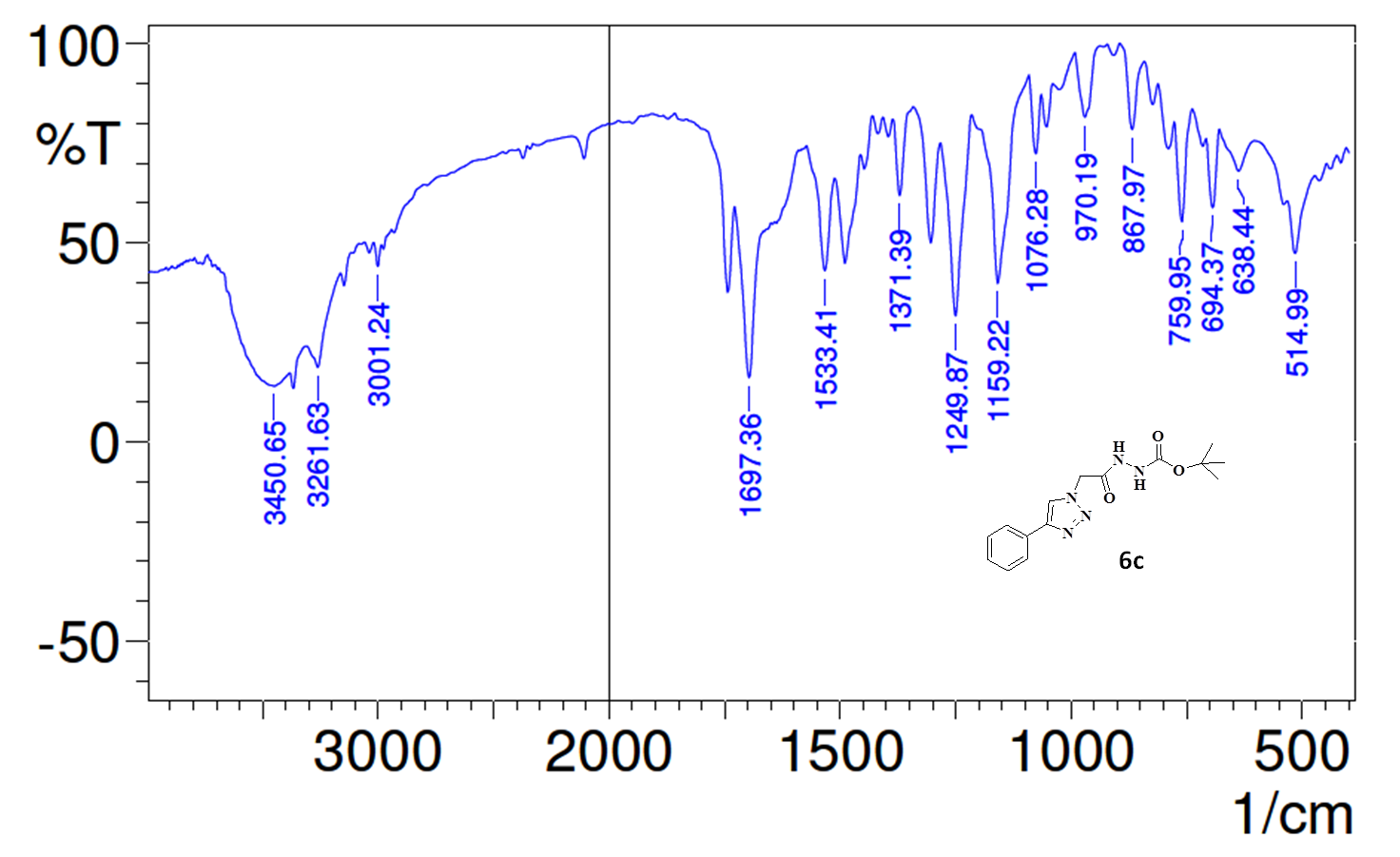
**

**4c**

**
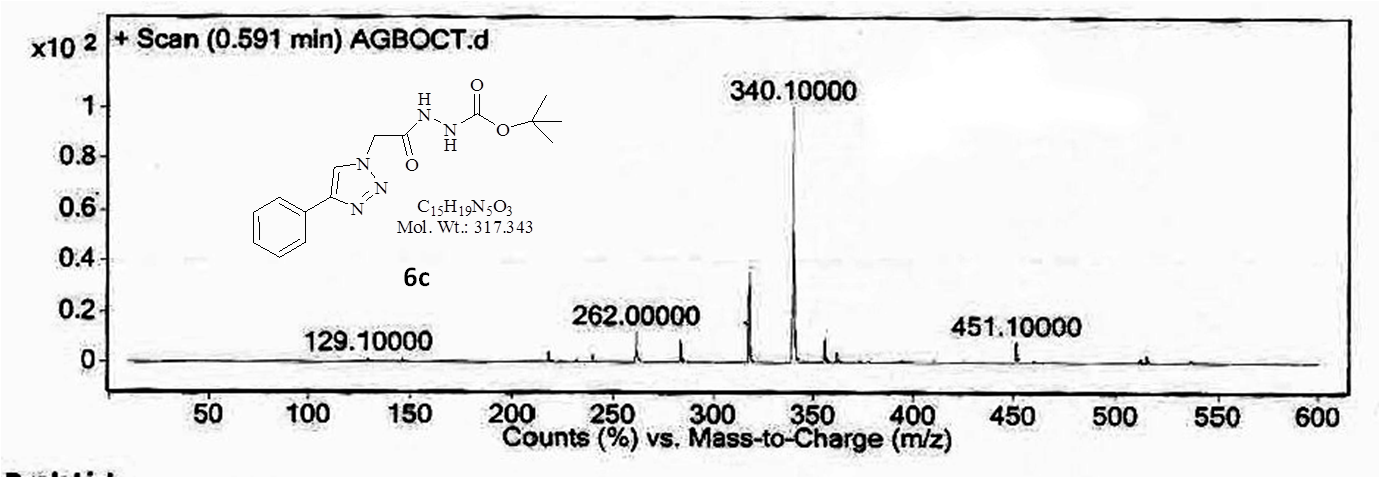
**

**4c**

**Figure 7 FTIR & Mass Spectrum of N'-[2-(4-Phenyl-[1,2,3]triazol-1-yl)-acetyl]-hydrazinecarboxylic acid tert-butyl ester (4c)**

**
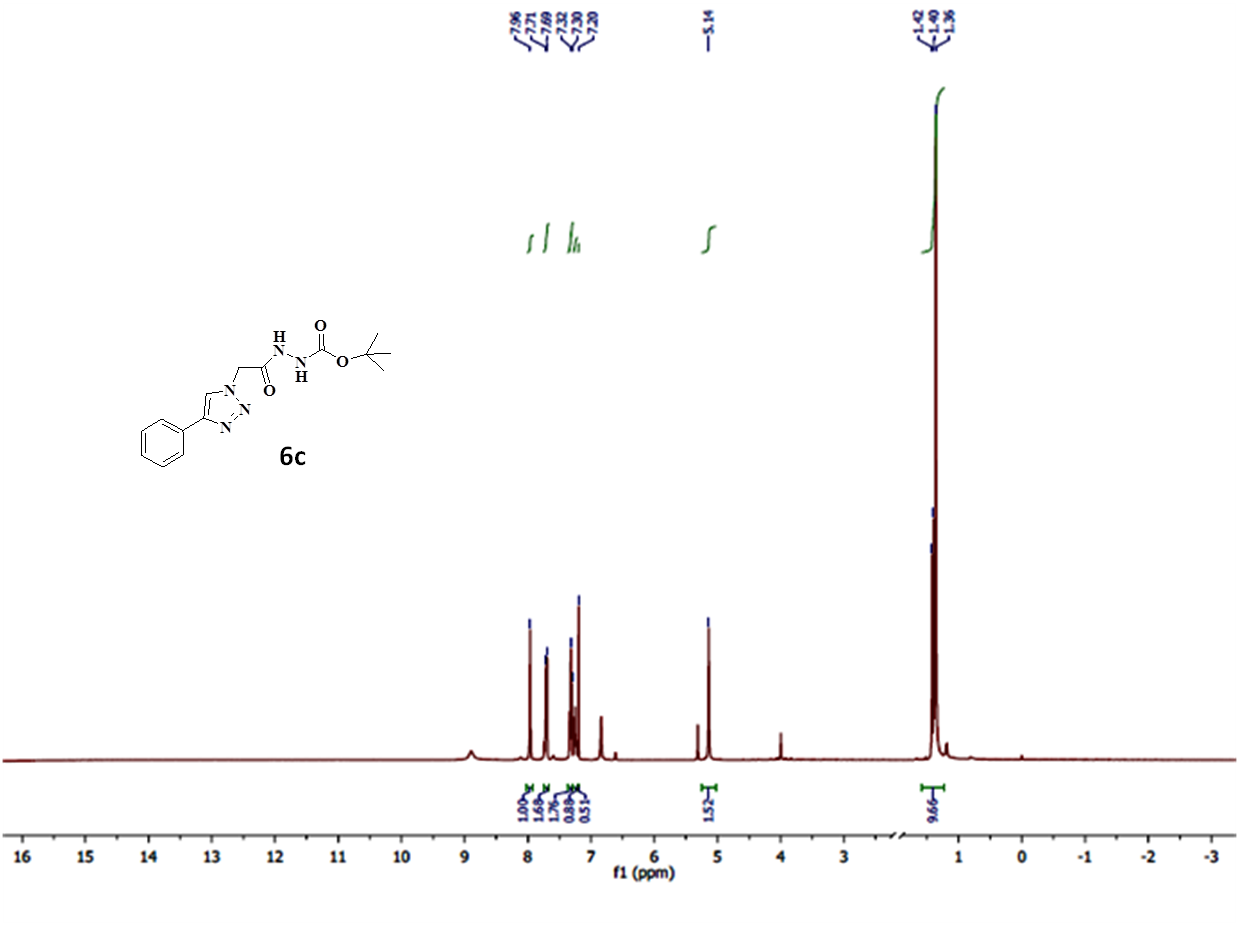
**

**4c**

**
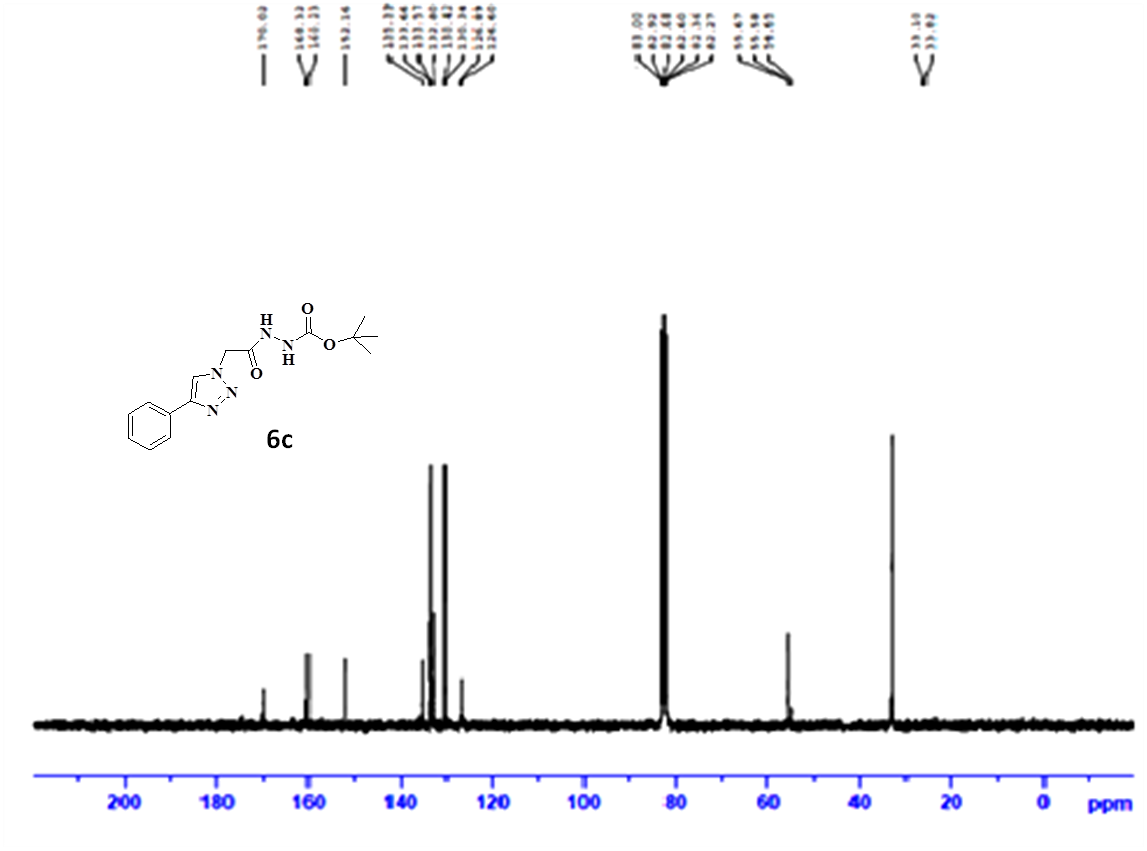
**

**4c**

**Figure 8: ^1^H-NMR and ^13^C-NMR Spectrum of N'-[2-(4-Phenyl-[1,2,3]triazol-1-yl)-acetyl]-hydrazinecarboxylic acid tert-butyl ester (6c)**

**2.5 2-(4-Phenyl-[1,2,3]triazol-1-yl)-N-pyrazin-2-yl-acetamide (5c)**

**
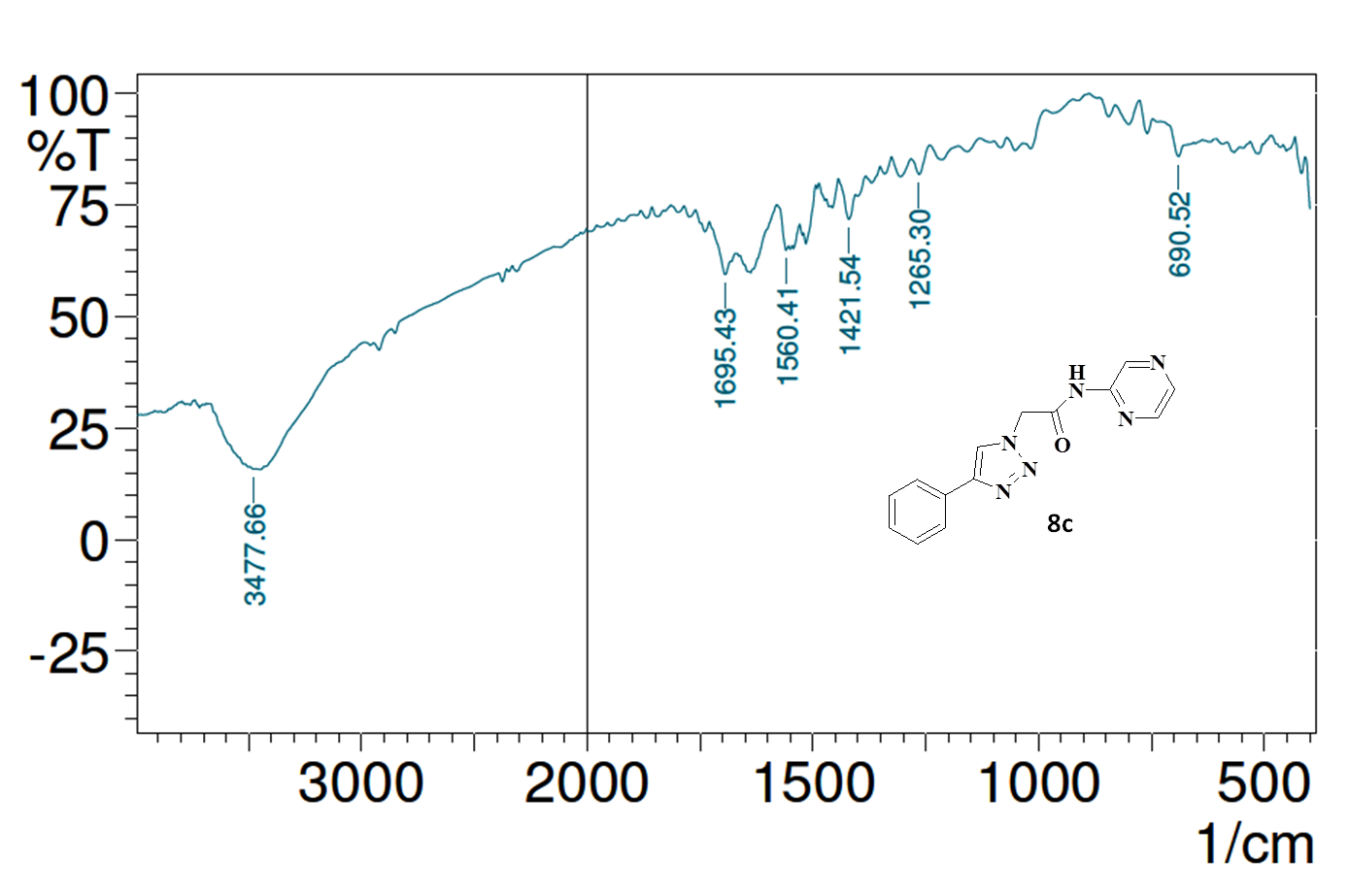
**

**5c**

**
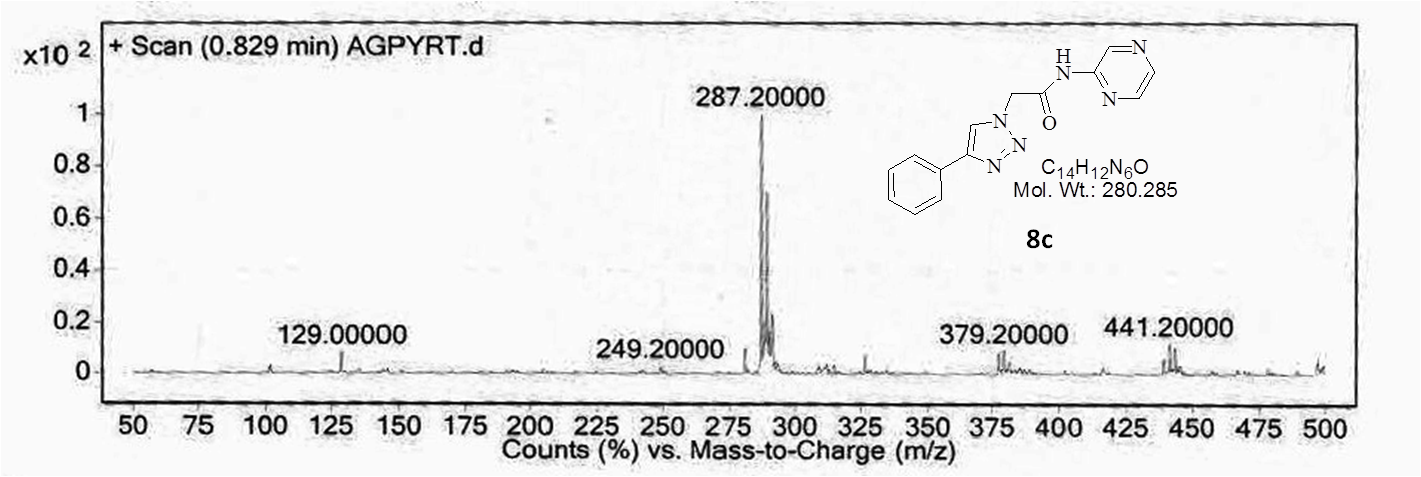
**

**5c**

**Figure 9 FTIR & Mass Spectrum of 2-(4-Phenyl-[1,2,3]triazol-1-yl)-N-pyrazin-2-yl-acetamide (8c)**

**
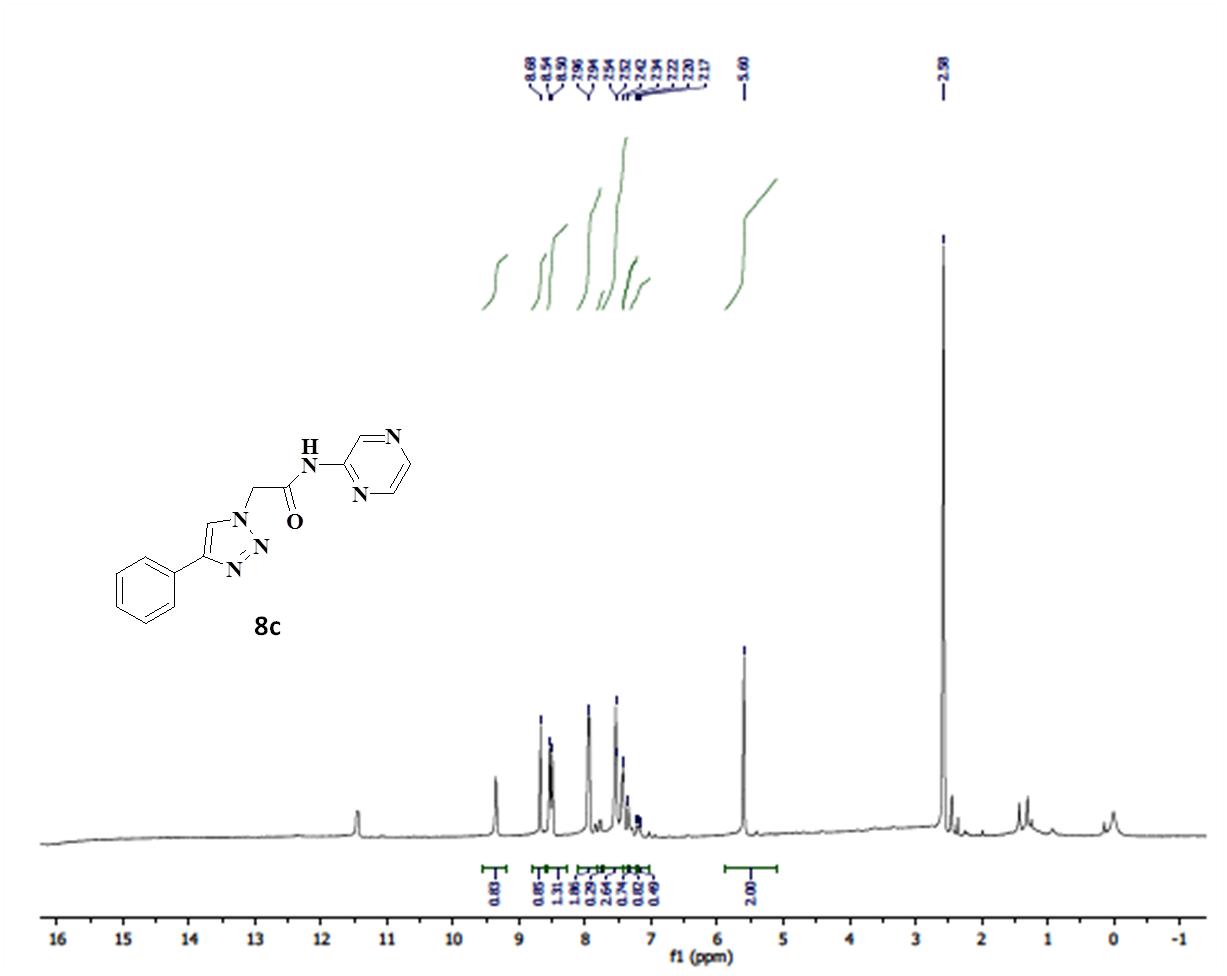
**

**5c**

**
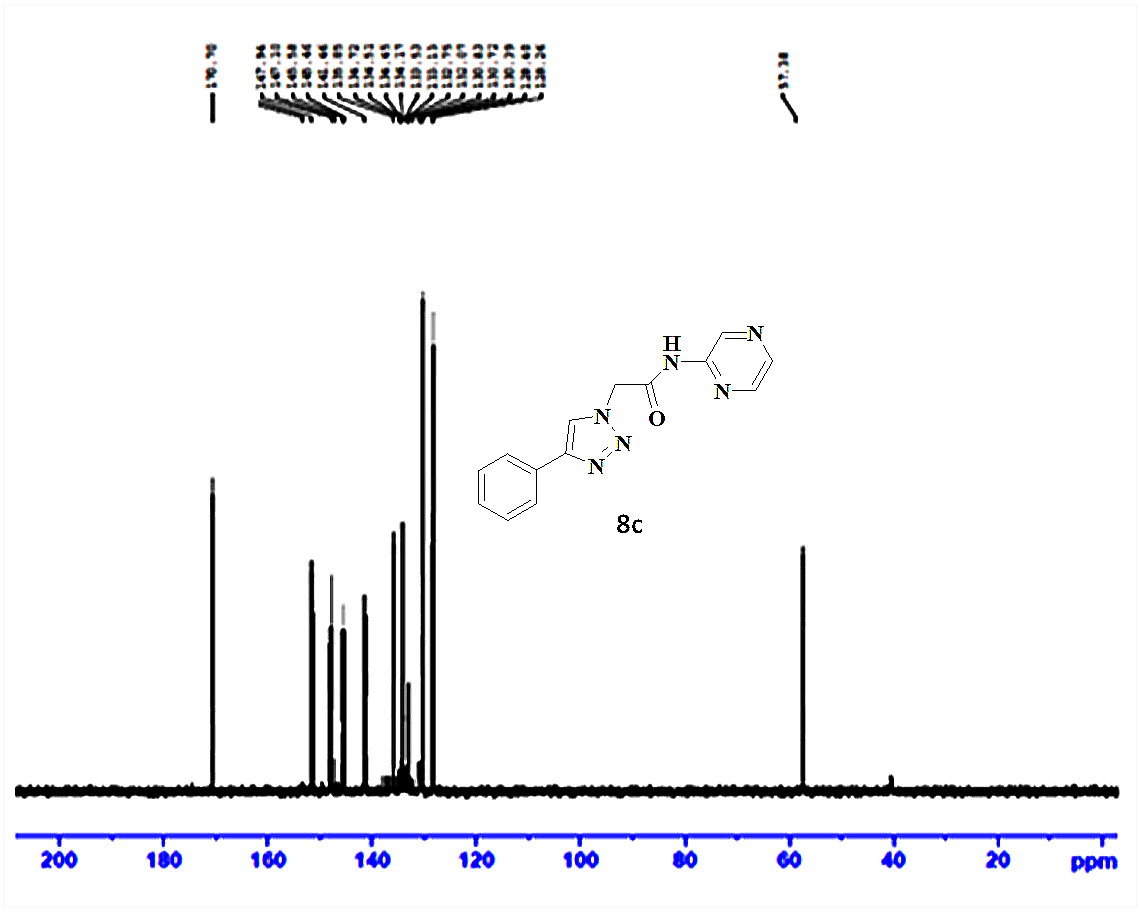
**

**5c**

**Figure 10. ^1^H-NMR spectrum of 2-(4-Phenyl-[1,2,3]triazol-1-yl)-N-pyrazin-2-yl-acetamide (52.5.10 4-Phenyl-1-(toluene-4-sulfonyl)-1H-[1,2,3]triazole (10c)**

**2.6 4-Phenyl-1-(toluene-4-sulfonyl)-1H-[1,2,3]triazole (6c)**

**
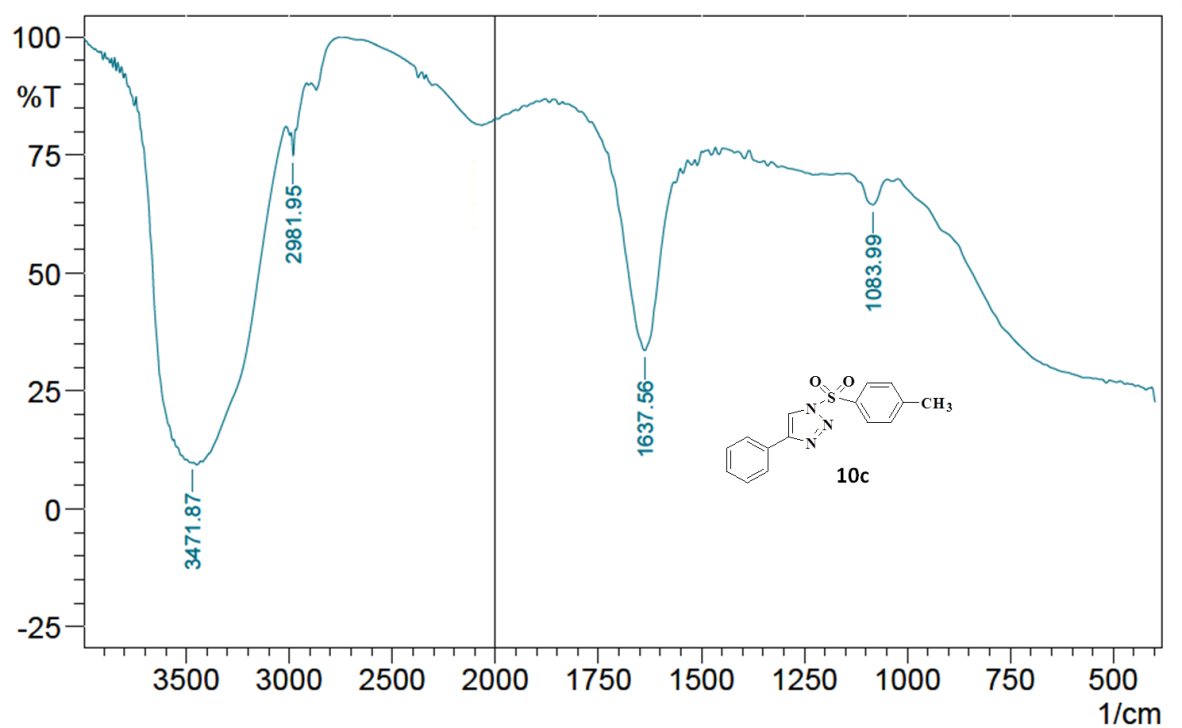
**

**6c**


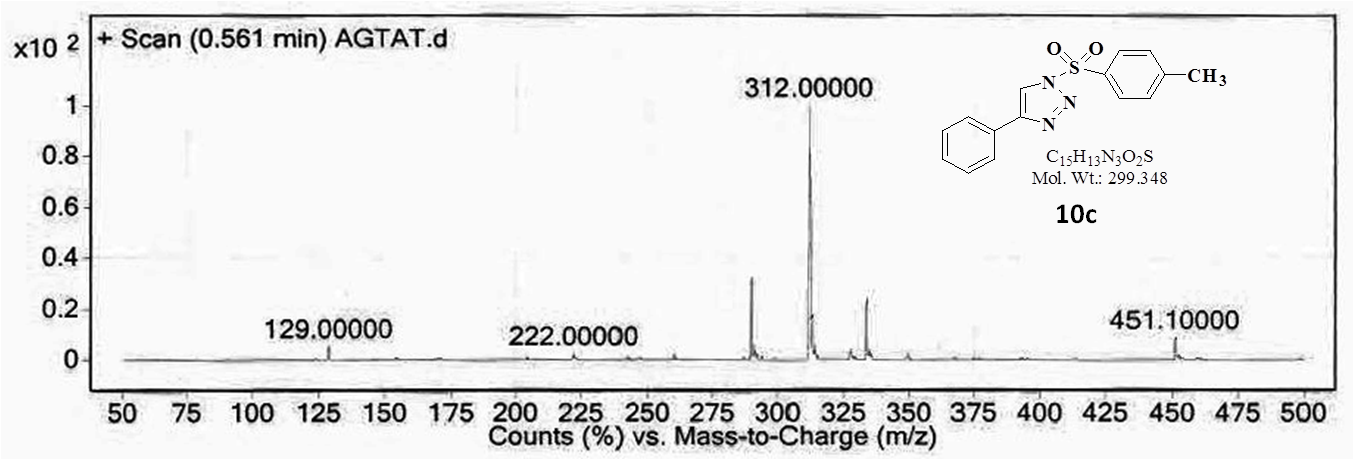


**6c**

**Figure 11. FTIR & Mass spectrum of 4-Phenyl-1-(toluene-4-sulfonyl)-1H-[1,2,3]triazole (6c)**

**
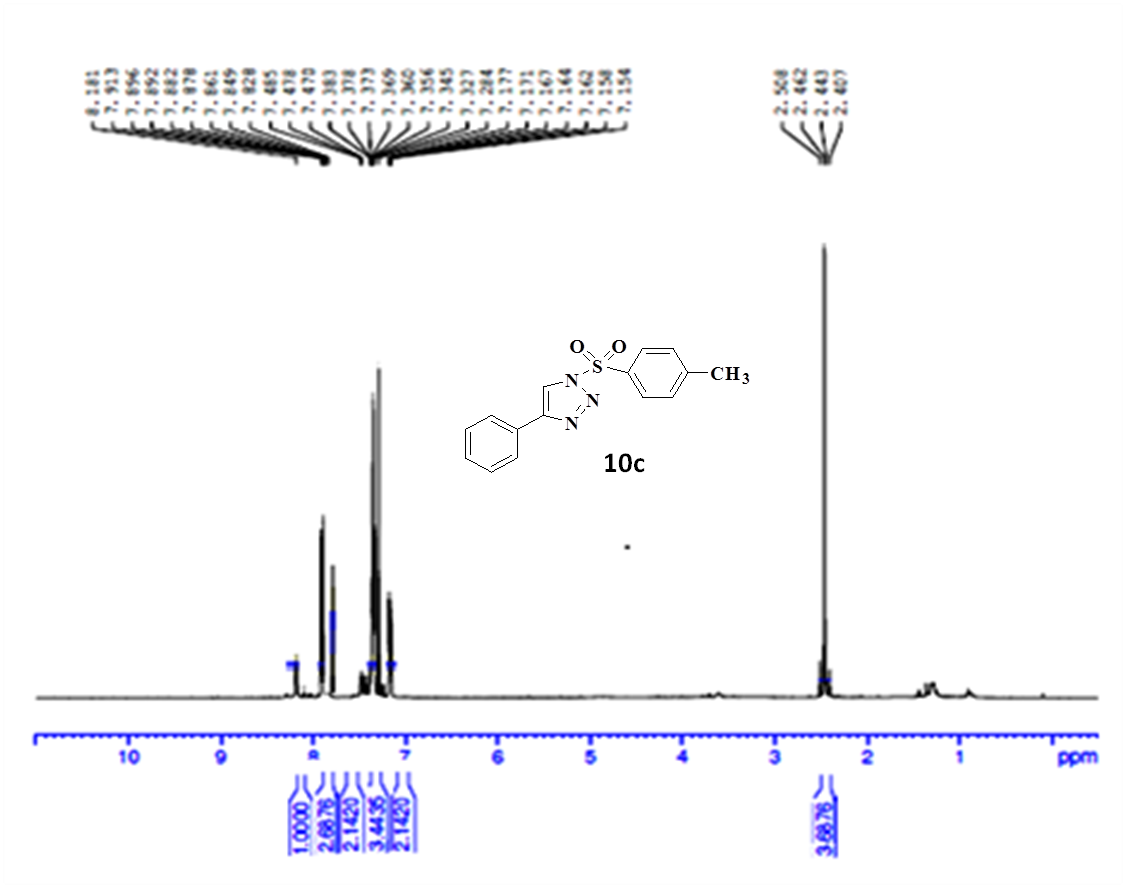
**

**6c**

**
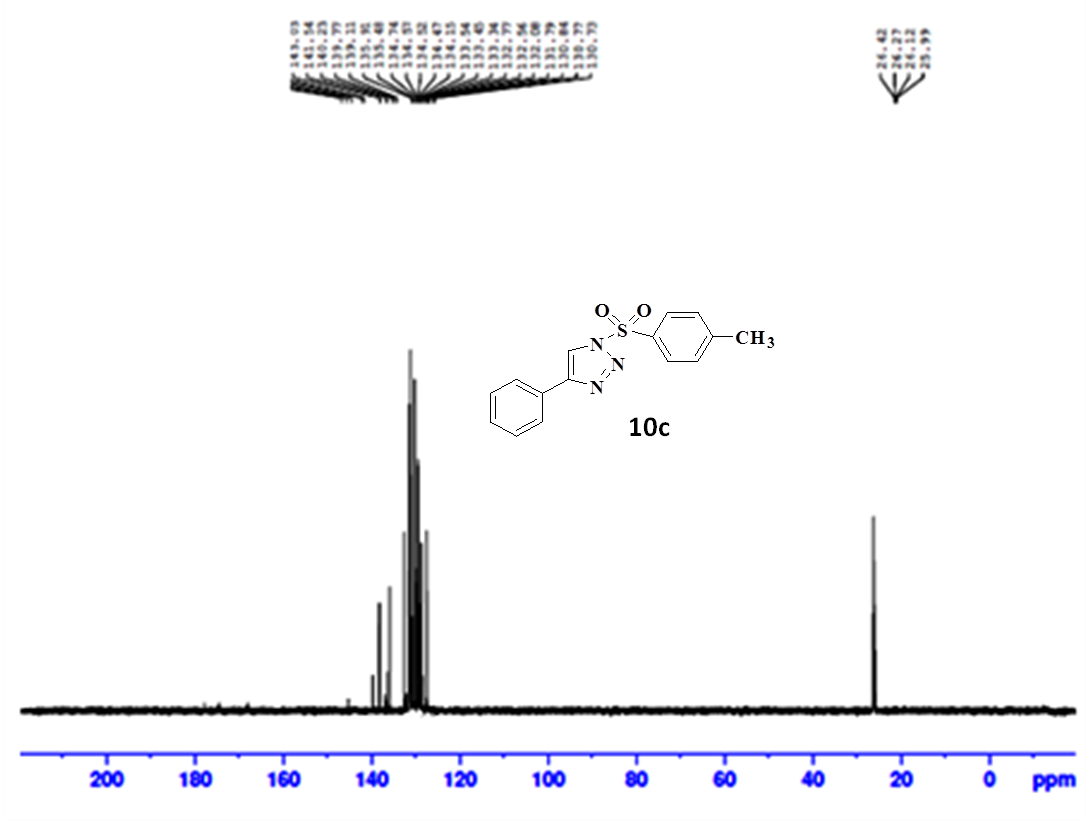
**

**6c**

**Figure 12. ^1^H-NMR & ^13^C-NMR Spectrum of 4-Phenyl-1-(toluene-4-sulfonyl)-1H-[1,2,3]triazole (6c)**

**3. 1-Methanesulfonyl-4-phenyl-1H-[1,2,3]triazole (7c)**

**
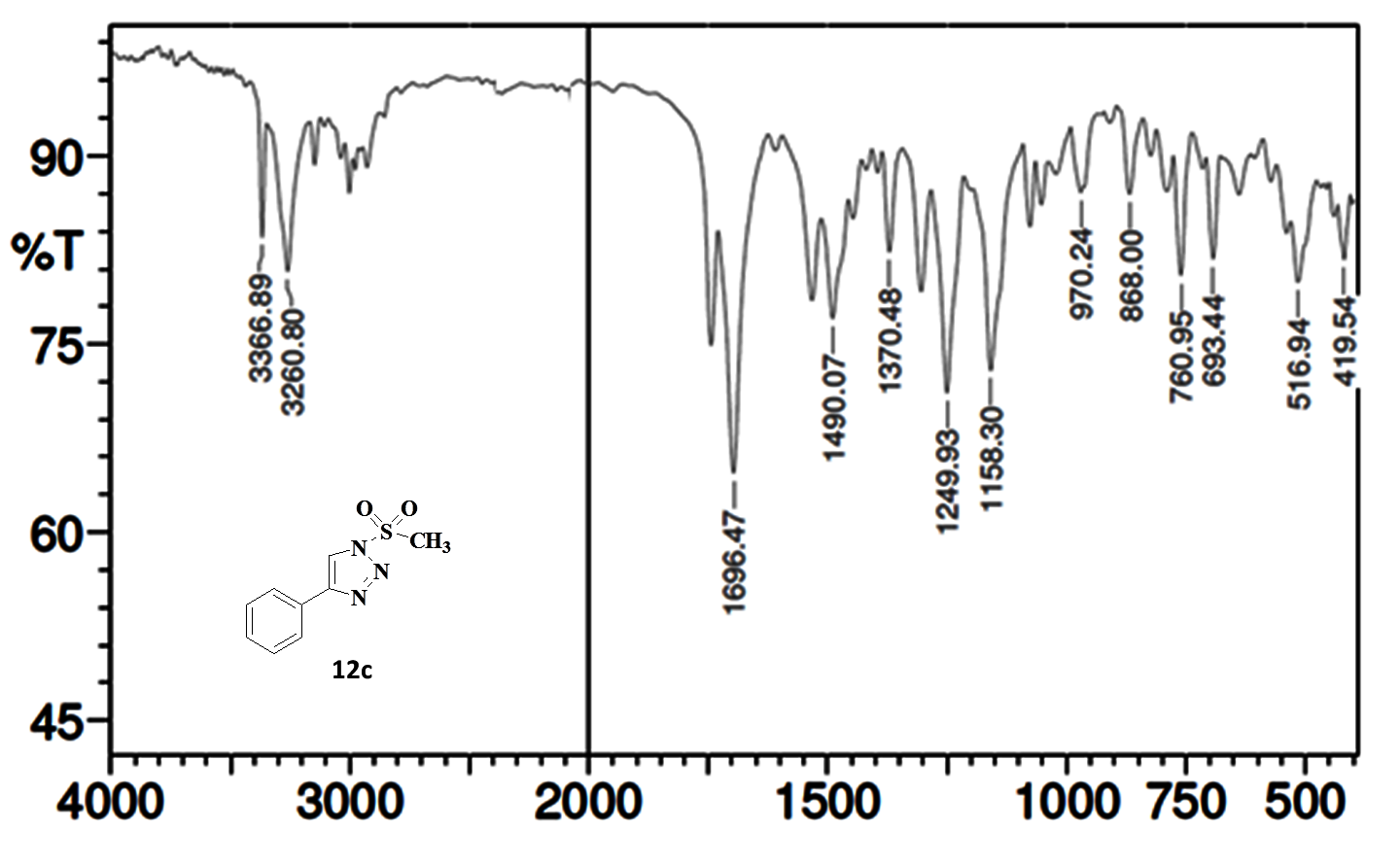
**

**7c**

**
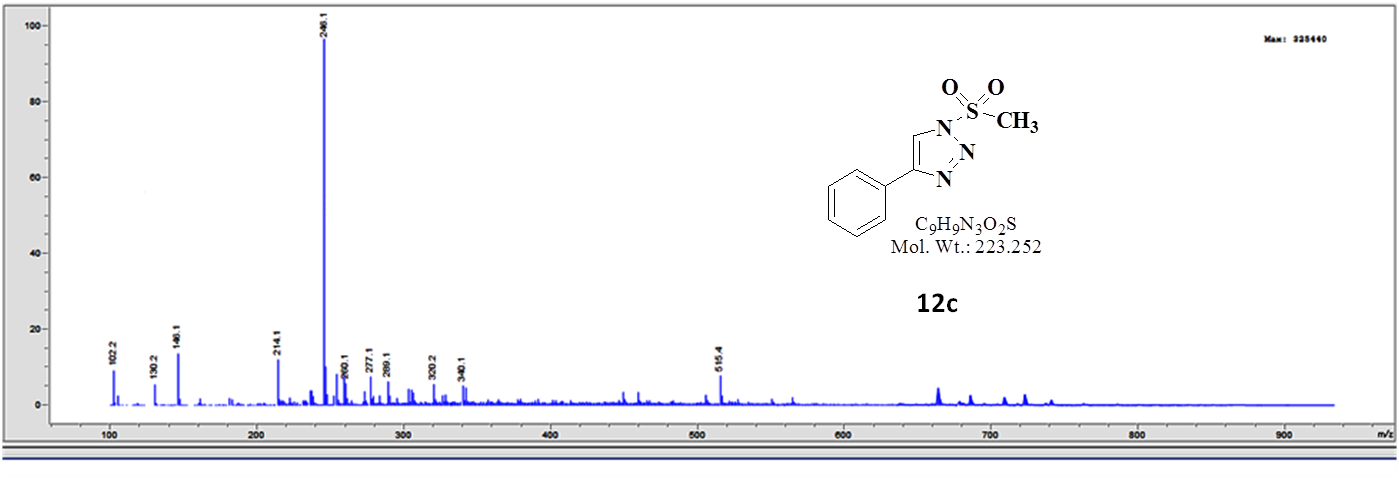
**

**7c**

**Figure 13. FTIR & Mass Spectrum of 1-Methanesulfonyl-4-phenyl-1H-[1,2,3]triazole (12c)**

**
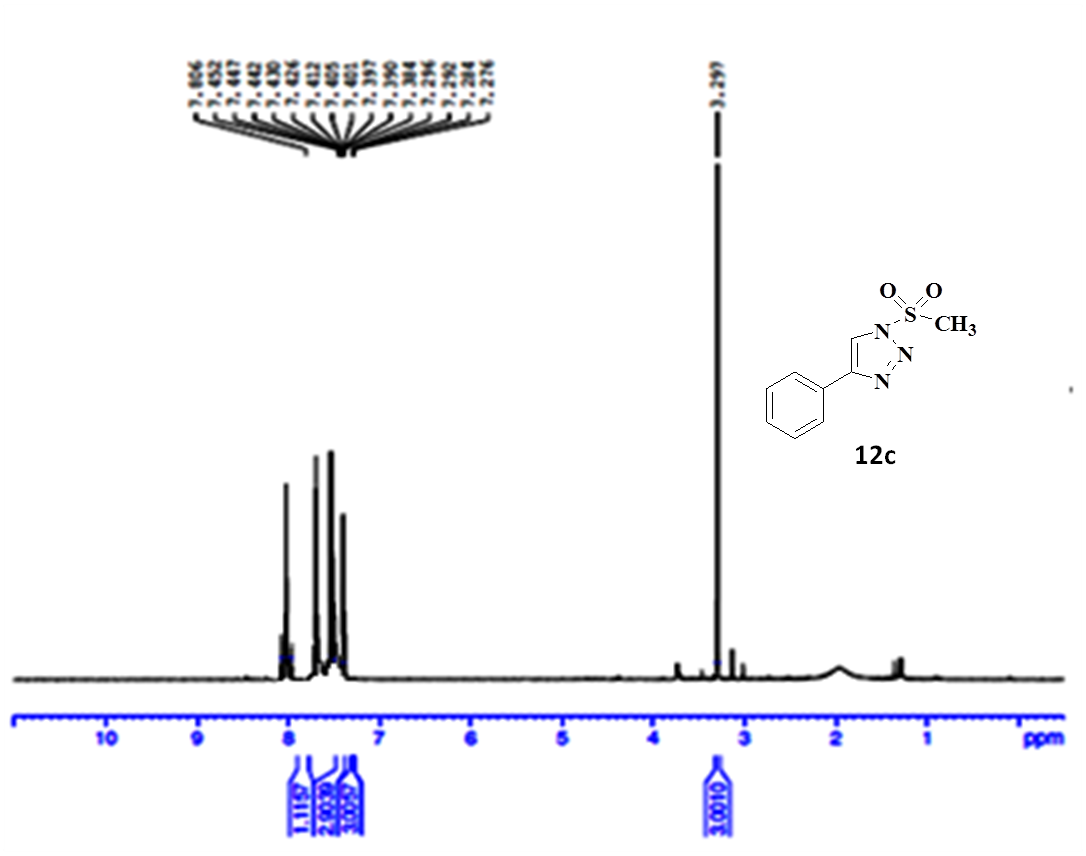
**

**7c**

**
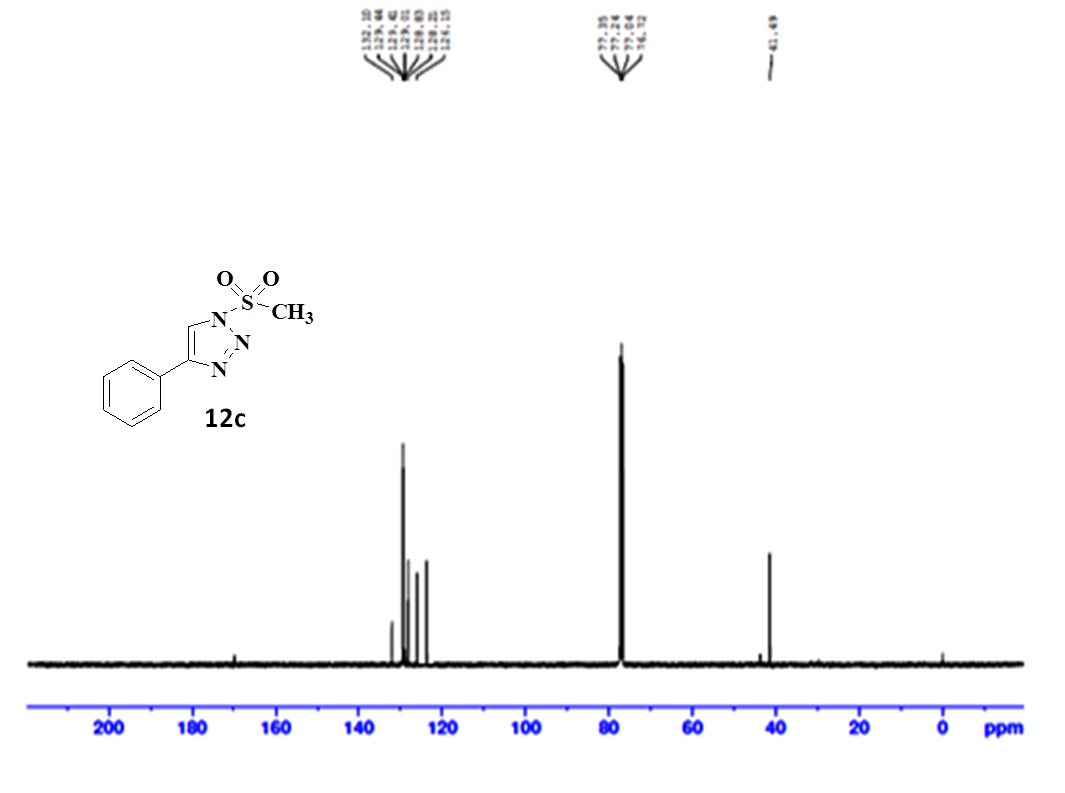
**

**7c**

**Figure 14. ^1^H-NMR & ^13^C-NMR Spectrum of 1-Methanesulfonyl-4-phenyl-1H-[1,2,3]triazole (7c)**

**2.8. Spectral Characterization of Cu(II)-Bis-cyclen complex.**

**
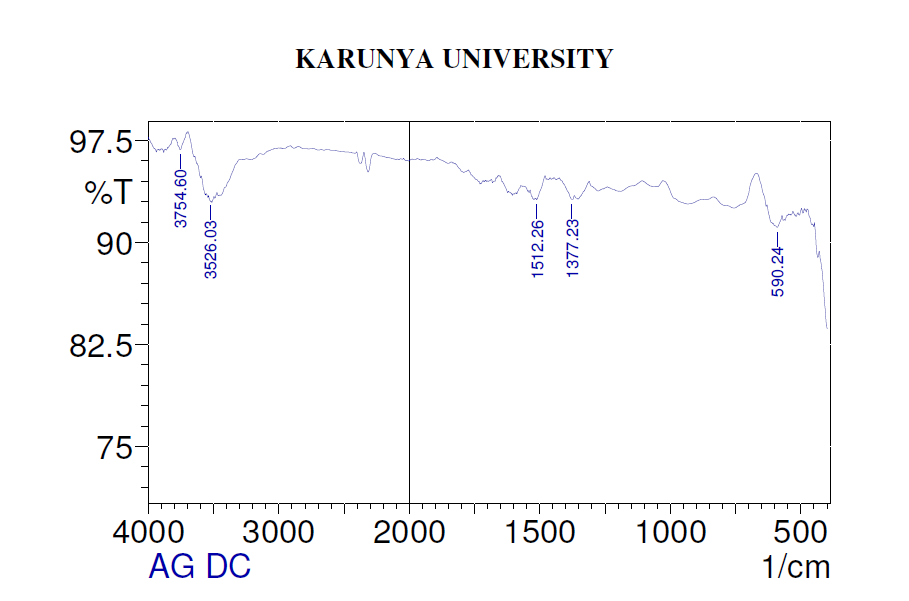
**

**Figure 15. FTIR & Mass Spectrum of Cu(II)-bis-cyclen-complex**

**References**

1. Tale, R.H., Gopula, V.B. and Toradmal, G.K., (2015). ‘Click’ ligand for ‘click’chemistry:(1-(4-methoxybenzyl)-1-H-1, 2, 3-triazol-4-yl) methanol (MBHTM) accelerated copper-catalyzed [3+ 2] azide–alkyne cycloaddition (CuAAC) at low catalyst loading. *Tetrahedron letters*, *56*(43), 5864-5869. doi:10.1016/j.tetlet.2015.09.010.
2. Wang, D., Li, N., Zhao, M., Shi, W., Ma, C. and Chen, B. (2010). Solvent-free synthesis of 1, 4-disubstituted 1, 2, 3-triazoles using a low amount of Cu(PPh_3_)_2_NO_3_ complex. *Green Chemistry*, *12*(12), 2120-2123. doi:10.1039/C0GC00381F.
3. Pourjavadi, A., Tajbakhsh, M., Farhang, M. and Hosseini, S.H. (2015). Copper-loaded polymeric magnetic nanocatalysts as retrievable and robust heterogeneous catalysts for click reactions. *New Journal of Chemistry*, *39*(6), 4591-4600. Doi:10.1039/C4NJ02134G
4. Candelon, N., Lastécouères, D., Diallo, A.K., Aranzaes, J.R., Astruc, D. and Vincent, J.M., (2008). A highly active and reusable copper (I)-tren catalyst for the “click” 1, 3-dipolar cycloaddition of azides and alkynes. *Chemical communications*, (6), 741-743.
5. Sarode, P.B., Bahekar, S.P. and Chandak, H.S., (2016). DABCO/AcOH jointly accelerated copper (I)-catalysed cycloaddition of azides and alkynes on water at room temperature. *Synlett*, *27*(19), 2681-2684.
